# Rotenone Induces a Neuropathological Phenotype in Cholinergic-like Neurons Resembling Parkinson’s Disease Dementia (PDD)

**DOI:** 10.1007/s12640-024-00705-3

**Published:** 2024-06-06

**Authors:** Daniela Giraldo-Berrio, Miguel Mendivil-Perez, Carlos Velez-Pardo, Marlene Jimenez-Del-Rio

**Affiliations:** Neuroscience Research Group, Medical Research Institute, Faculty of Medicine, University of Antioquia (UdeA), Calle 70 No. 52-21, and Calle 62 # 52-59, Building 1, Room 412, Medellin, Antioquia Colombia

**Keywords:** Alzheimer’s disease, Alpha-synuclein, E280A, Parkinson, Presenilin, Mutation, Rotenone

## Abstract

Parkinson’s disease with dementia (PDD) is a neurological disorder that clinically and neuropathologically overlaps with Parkinson’s disease (PD) and Alzheimer’s disease (AD). Although it is assumed that alpha-synuclein ($\mathrm{\alpha }$-Syn), amyloid beta (A$\upbeta$), and the protein Tau might synergistically induce cholinergic neuronal degeneration, presently the pathological mechanism of PDD remains unclear. Therefore, it is essential to delve into the cellular and molecular aspects of this neurological entity to identify potential targets for prevention and treatment strategies. Cholinergic-like neurons (ChLNs) were exposed to rotenone (ROT, 10$\upmu$M) for 24 h. ROT provokes loss of $\Delta \mathrm{\Psi m}$, generation of reactive oxygen species (ROS), phosphorylation of leucine-rich repeated kinase 2 (LRRK2 at Ser^935^) concomitantly with phosphorylation of $\mathrm{\alpha }$ -synuclein ($\mathrm{\alpha }$-Syn, Ser^129^), induces accumulation of intracellular A$\upbeta$ (iA$\upbeta$), oxidized DJ-1 (Cys^106^), as well as phosphorylation of TAU (Ser^202^/Thr^205^), increases the phosphorylation of c-JUN (Ser^63^/Ser^73^), and increases expression of proapoptotic proteins TP53, PUMA, and cleaved caspase 3 (CC3) in ChLNs. These neuropathological features resemble those reproduced in presenilin 1 (PSEN1) E280A ChLNs. Interestingly, anti-oxidant and anti-amyloid cannabidiol (CBD), JNK inhibitor SP600125 (SP), TP53 inhibitor pifithrin-$\mathrm{ \alpha }$ (PFT), and LRRK2 kinase inhibitor PF-06447475 (PF475) significantly diminish ROT-induced oxidative stress (OS), proteinaceous, and cell death markers in ChLNs compared to naïve ChLNs. In conclusion, ROT induces p-$\mathrm{ \alpha }$ -Syn, iA$\upbeta$, p-Tau, and cell death in ChLNs, recapitulating the neuropathology findings in PDD. Our report provides an excellent in vitro model to test for potential therapeutic strategies against PDD. Our data suggest that ROT induces a neuropathologic phenotype in ChLNs similar to that caused by the mutation PSEN1 E280A.

**Supplementary Information:**

The online version contains supplementary material available at 10.1007/s12640-024-00705-3.

## Introduction

Parkinson’s disease (PD) is a progressive chronic neurologic disorder clinically characterized by slowing of movements in the limbs, face, walking or overall body (bradykinesia), resting tremor which typically stops when the patient is active or moving, rigidity or stiffness in the arms, legs or trunk (Jankovic 2008; Bloem et al. 2021). Alzheimer’s disease (AD) is also a common progressive disorder beginning with mild memory loss leading to typical features of dementia such as severe impairments in thought, memory, and language (Knopman et al. 2021). While PD is the consequence of dramatic loss of dopaminergic (DAergic) neurons in the ventral part of the pars compacta in the substantia nigra and deficit of dopaminergic innervation of striatum (Dickson 2012), AD is caused by the losses of forebrain cholinergic neurons of the nucleus basalis of Meynert (Ch4) and cholinergic projection from the Medial septum nucleus (Ch1) to hippocampus (Liu et al. 2015; Pepeu and Grazia Giovannini 2017). Intriguingly, Parkinson’s disease with dementia (PDD) is a neurological disorder that overlaps with PD and AD (Irwin et al. 2013). By definition, PDD is a clinical condition wherein patients with diagnosis of PD goes together with cognitive dysfunction (e.g., deficits in attention, executive functioning, visuospatial processing), neuropsychiatric symptoms (e.g., hallucinations, depression), memory loss and dementia (Goetz et al. 2009; Vasconcellos and Pereira 2015; Phillips et al. 2023). Pathologically, PDD is characterized by severe burden of Lewy bodies, which are composed of the pathological PD-associated protein $\mathrm{\alpha }$ -synuclein ($\mathrm{\alpha }$-Syn) and Lewy neurites, as well as extensive diffuse amyloid-β‑reactive plaque pathology, Tau-reactive diffuse threads and neurofibrillary tangles (NFT), which are AD-associated pathological markers (Smith et al. 2019; Kouli et al. 2020). Although it is supposed that $\mathrm{\alpha }$- Syn, A$\upbeta$, and Tau might synergistically induce cholinergic and DAergic neuronal degeneration (Kim 2023), presently the pathological mechanism of PDD remains unclear. Therefore, it is essential to delve into the cellular and molecular aspects of this neurological entity to identify potential targets for the prevention and treatment strategies (Han et al. 2021).

Rotenone (ROT, PubChem CID 6758), a naturally occurring organic compound found in the roots of the *Derris* (Sae-Yun et al. 2006; Zubairi et al. 2014) and *Lonchocarpus* (Fang and Casida 1999) plant species, is used worldwide due to its broad spectrum insecticidal (Zhang et al. 2022a), acaricidal and pesticide properties (http://www.chm.bris.ac.uk/motm/rotenone/; available in November, 2023). Importantly, ROT induces specific degeneration of DAergic neurons in vitro and in vivo (Lawana and Cannon 2020)*,* which intrinsically deteriorate in PD (Giguère et al. 2018). Mechanistically, ROT acts as a strong inhibitor of complex I of the mitochondrial respiratory chain (Read et al. 2021) via inhibition of electron transfer from the iron-sulfur centers in complex I to ubiquinone, leading to a blockade of the I_Q_ site (Schiller and Zickermann 2022), and over-reduction of complex I causes electrons to leak and produce reactive oxygen species (ROS) such as superoxide anion radical (O_2_^.−^). This radical can dismutate into non-radical reactive hydrogen peroxide, H_2_O_2_ (Li et al. 2003; Mailloux 2015). In turn, this last compound, via signaling mechanisms (Marinho et al. 2014; Antunes and Brito 2017), induces regulated cell death apoptosis (Velez-Pardo and Jimenez-Del-Rio 2020). Although, the effect of ROT appears tissue-specific, it has also been demonstrated that ROT induces cell death of non-catecholaminergic neurons such as cholinergic neurons (ChNs) in an organotypic co-culture brain slice model (Ullrich and Humpel 2009). This last observation suggests that hippocampal Lewy pathology might be associated with cholinergic degeneration in PD with cognitive decline (Liu et al. 2019; Aarsland et al. 2021). These may explain why some brains from PDD patients present AD features such as A$\upbeta$ plaques and tau containing NFT (Irwin et al. 2013; Jellinger 2023). These observations suggest that $\mathrm{\alpha }$ -Syn, A$\upbeta$, and Tau might together induce neuronal degeneration. In line with this view, it has been shown that ROT induced $\mathrm{\alpha }$-Syn and A$\upbeta$ aggregation, as well as increased hyperphosphorylation of Tau in cultured cells from hippocampus, locus coeruleus and substantia nigra of newborn Lewis rats (Chaves et al. 2010). Moreover, ROT triggered a cerebral tauopathy in rats (Höglinger et al. 2005). However, the mechanism by which ROT induce co-existence of $\mathrm{\alpha }$-Syn, A$\upbeta$, and Tau is unknown. Furthermore, the mechanism by which ROT produces proteinopathy in cholinergic neurons is yet unknown. Although ROT has extensively been used as model neurotoxin to disclose the molecular events of PD (Ke et al. 2021), currently there are not in vitro model of PDD available.

Mesenchymal stromal cells (MSCs) derived from umbilical cord Warton’s Jelly (UC-WJ) are multipotent cells that have the potential to differentiate into neuroectodermal cell lineage (Dominici et al. 2006; Viswanathan et al. 2019). Our laboratory has used WJ-MSCs to recapitulate the neuropathological features of familial AD (FAD) due to a mutation in presenilin 1 (PSEN1) E280A (Soto-Mercado et al. 2020). Indeed, cultured MSCs bearing the wild-type (WT) and variant PSEN1 E280A in *Cholinergic-N-Run* medium (Mendivil-Perez et al. 2019) transdifferentiated into cholinergic-like neurons (ChLNs). Notably, PSEN1 E280A ChLNs but not WT PSEN1 ChLNs showed accumulation of intracellular amyloid precursor protein fragments (iAPP$\upbeta$f/ iA$\upbeta$), and displayed phosphorylation of protein TAU (p-TAU at residues Ser^202^/Thr^205^). Also, the variant ChLNs E280A presented oxidation of stress sensor DJ-1Cys^106^-SH into DJ-1Cys^106^-SO_3_, phosphorylation of c-JUN at Ser^63^/Ser^73^, and detection of dichlorofluorescein (DCF)-positive cells as evidenced of generation of ROS such as H_2_O_2_, oxidative stress (OS), concomitant loss of the $\Delta \mathrm{\Psi m}$, DNA fragmentation, and Ca^2+^ flux dysregulation (Soto-Mercado et al. 2020). Despite these advances, no chemical neurotoxin-induced model of FAD has yet been revealed.

Given that PDD appears neuropathologically to recapitulate AD and PD, we hypothesize that ROT can replicate both FAD and PD cellular hallmarks in ChLNs, which represent an excellent model to study PDD. We have treated ChLNs derived from WJ-MSCs with ROT (1, 5, 10 $\upmu$M) for 24 h. For comparative and validation purposes, we used WJ-MSC-derived PSEN1 E280A ChLNs. We found that (i) ROT induces generation of ROS and H_2_O_2_, loss of $\Delta \mathrm{\Psi m}$, oxidized DJ-1, accumulation of iA$\upbeta$, p-Ser^202^/Thr^205^ TAU, and cell death happens in ChLNs, as it appears in PSEN1 E280A ChLNs FAD; (ii) ROT induces concomitant p-Ser^935^ LRRK2 and p-Ser^129^-$\mathrm{ \alpha }$-Syn in ChLNs. Outstandingly, we report for the first time that p-LRRK2 and p-$\mathrm{ \alpha }$-Syn also endogenously appear in PSEN1 E280A ChLNs; (iii) ROT increases the phosphorylation of c-JUN at residues Ser^63^/Ser^73^ and the expression of TP53, PUMA and pro-apoptotic marker cleaved caspase 3 (CC3) in ChLNs. A similar profile of positive protein markers was also observed in PSEN1 E280A ChLNs; (iv) ROT impairs ACh-induced transient Ca^2+^ influx in ChLNs to a similar extend as observed in PSEN1 E280A ChLNs. Interestingly, ChLNs co-treated with ROT and anti-amyloidogenic and antioxidant cannabidiol, JNK inhibitor SP600125, and LRRK2 inhibitor PF-06447475 significantly blunted A$\upbeta$, oxDJ-1, p-$\mathrm{ \alpha }$-Syn, p-TAU, and CC3, respectively. Given that ROT and iA$\upbeta$ trigger mitochondrial dysfunction, and produce H_2_O_2_, they may be an important etiopathogenic factor involved not only in AD and PD (Velez-Pardo and Jimenez-Del-Rio 2020) but also in PDD. Therefore, mitochondria, LRRK2, and H_2_O_2_ might be targets for therapeutic treatment for PDD (Macdonald et al. 2018; Abrishamdar et al. 2023).

## Materials and Methods

### Transdifferentiation of Mesenchymal Stromal Cell into Cholinergic-like Neurons

ChLN differentiation was performed according to (Mendivil-Perez et al. 2019). Briefly, the WT (TBC# WJMSC-19) and PSEN1 E280A (TBC# WJMSC-24) were obtained from the Neuroscience Tissue Bank-UdeA. MSCs were seeded at 1–1.5 × 10^4^ cells/cm^2^ in laminin-treated culture plates for 24 h in regular culture medium (RCm). The medium was removed, and cells were incubated in cholinergic differentiation medium (*Cholinergic-N-Run* medium, hereafter *Ch–N-Rm*) containing DMEM/F-12 media 1:1 Nutrient Mixture (Gibco cat# 10,565,018; 1204 N Western St, Suite C, Amarillo, TX, USA), 10 ng/mL basic fibroblast growth factor (bFGF) recombinant human protein (Gibco Cat# 13,256,029), 50 µg/mL sodium heparin (Hep, Sigma-Aldrich cat# H3393; 3050 Spruce Street, St. Louis, MO 63103, USA), 0.5 µM all-trans retinoic acid, 50 ng/mL sonic hedgehog peptide (SHH, Sigma cat# SRP3156) and 1% FBS at 37 °C for 7 days. After this process of transdifferentiation, the cells were labeled as WT PSEN1 or PSEN1 E280A ChLNs. Since *Ch–N-Rm* contains several factors (e.g., growth factors) that might interfere with the experiment interpretation and measurements, WT PSEN1 and PSEN1 E280A ChLNs (obtained after 7 days in *Ch–N-Rm*) were left in a regular culture medium (RCm) for 4 additional days of post transdifferentiation (Mendivil-Perez et al. 2019).

### Assay Protocol

An initial rotenone (ROT) screening was performed at least twice in triplicate including 1, 5, and 10 μM final concentrations. Subsequently, 10 μM ROT was established as an optimal concentration for further experiments. For analyses, ChLNs were divided into two groups: (i) untreated WT PSEN1; and (ii) WT PSEN1 treated with 10 μM ROT. Chemical inhibition assay was performed by pre-incubating WT PSEN1 in absence or presence of cannabidiol (CBD; 10 μM, Mendivil-Perez et al. 2023), the anthrapyrazolone JNK inhibitor SP600125 (SP; 1 μM, Soto-Mercado et al. 2020), cell-permeable chemical inhibitor of p53 pifithrin-$\mathrm{ \alpha }$(PFT-$\mathrm{ \alpha }$; 50 nM, Velez-Pardo et al. 2002), and the potent and selective LRRK2 inhibitor PF-06447475 (PF475; 1 μM, Mendivil-Perez et al. 2016) for 30 min previous to ROT exposure. The PSEN1 E280A ChLNs were used for comparative and validation purposes.

### Immunofluorescence Analysis

The analysis of Alzheimer’s disease-, oxidative stress- and cell death-related markers, was exactly performed as described elsewhere (Soto-Mercado et al. 2020). Briefly, the cells treated under different conditions were fixed with 4% paraformaldehyde for 20 min, followed by Triton X-100 (0.1%) permeabilization and 10% bovine serum albumin (BSA) blockage. Cells were incubated overnight with primary antibodies against the first 2 amino acids of the A$\upbeta$ peptide amino N-terminus, namely the A$\upbeta$4 1E8 antibody (1:500; clone 1E8 cat# MABN639, Millipore, 3050 Spruce Street, St. Louis, MO 63304, USA), phospho-TAU (p-Tau, 1:500, Ser^202^/Thr^205^, cat# MN1020 (AT8); and primary antibodies against oxidized DJ-1 (1:500; ox(Cys^106^)DJ-1; spanning residue C^106^ of human PARK7/DJ1; oxidized to produce cysteine sulfonic (SO_3_) acid; cat # ab169520, Abcam). To assess cell death, we used primary antibodies against PUMA conjugated with Alexa Fluor 488 (1:500; PUMA, sc-377015 AF488, Santa Cruz Biotechnology), p53 conjugated with Alexa Fluor 594 (1:500; cat# sc-126 AF594, Santa Cruz Biotechnology), phospho-c-Jun conjugated with Alexa fluor 594 (1:500; c-Jun (S^63^/^73^) cat# sc-822 AF594, Santa Cruz Biotechnology), and caspase-3 fluorescent probe (1:500; AM- DEV-FMK- caspase 3, V35118). To evaluate α-synuclein, we used primary antibodies against total α-synuclein (1:500; clone Syn211 cat# S5566, Sigma Aldrich), and phosphorylated α-synuclein (1:500; phosphoS^129^; cat# ab51253, Abcam). After exhaustive rinsing, we incubated the cells with secondary fluorescent antibodies (to identify non-conjugated antibodies reactions; DyLight 488 and 594 horse anti-rabbit, -goat and -mouse, cat DI 1094, DI 3088, and DI 2488, respectively) at 1:500. The nuclei were stained with 1 μM Hoechst 33,342 (Life Technologies), and images were acquired on an Axiovert coupled to Axicam miscoscope.

### Flow Cytometry Analysis

After each treatment, cells were detached using trypsin and centrifuged for 10 min at 2000 rpm. Then, cells were washed with PBS and fixed with cold ethanol (96%) overnight. Cells were washed two times with PBS and permeabilized with 0.2% Triton X-100 plus 1.5% bovine serum albumin (BSA) for 30 min. Cells were incubated overnight with primary antibodies against the first 2 amino acids of the A$\upbeta$ peptide amino N-terminus, namely the A$\upbeta$4 1E8 antibody (1:500; clone 1E8 cat# MABN639, Millipore, 3050 Spruce Street, St. Louis, MO 63304, USA), phospho-TAU (AT8; 1:200), oxidized DJ-1 (1:200), PUMA (1:200), p53 (1:200), phospho-c-Jun (1:200; c-Jun (S^63^/^73^), caspase-3 (1:200), total α-synuclein (1:200)and phosphorylated α-synuclein (1:200). After exhaustive rinsing, we incubated the cells with secondary fluorescent antibodies (DyLight 488 and 594 horse anti-rabbit, -goat and -mouse, cat DI 1094, DI 3088, and DI 2488, respectively) at 1:500. Fluorescence analysis was performed on a BD LSRFortessa II flow cytometer (BD Biosciences). Cells without primary antibodies served as a negative control. For assessment, 10,000 events and quantitative data and figures were obtained using FlowJo 7.6.2 Data Analysis Software (TIBCO® Data Science). Events analysis was performed by determining the cell population (Forward Scatter analysis, Y axis) that exceeded the basal fluorescence (488 nm or 594 nm, X axis) of the negative control. Accordingly, density plots or histograms were created from event analysis.

### Evaluation of Intracellular Reactive Oxygen Species (e.g., Hydrogen Peroxide, H_2_O_2_) by Fluorescence Microscopy

To assess the levels of intracellular ROS (H_2_O_2_), we used 2′,7′-dichlorofluorescein diacetate (5 μM, DCFH_2_-DA; Invitrogen) according to Soto-Mercado et al. 2020. ChLNs were left in RCm for 4 days. Then, the cells (5 × 10^3^) were incubated with the DCFH_2_-DA reagent for 30 min at 37 °C in the dark. Cells were then washed, and DCF fluorescence intensity was determined by analysis of fluorescence microscopy images (Lichtman and Conchello 2005). The assessment was repeated three times in independent experiments. The nuclei were stained with 0.5 µM Hoechst 33,342 staining compound. The assessment was repeated three times in independent experiments blind to the experimenter.

### Evaluation of Intracellular Hydrogen Peroxide (H_2_O_2_) by Flow Cytometry

To assess the levels of intracellular ROS (H_2_O_2_), we used 2′,7′-dichlorofluorescein diacetate (5 μM, DCFH_2_-DA; Invitrogen) according to Soto-Mercado et al. 2020. ChLNs were left in RCm for 4 days. Then, the cells (1 × 10^4^) were incubated with the DCFH_2_-DA reagent for 30 min at 37 °C in the dark. Cells were then washed, and DCF fluorescence was determined using an LSRFortessa (BD Biosciences). The assessment was repeated 3 times in independent experiments. Quantitative data and figures were obtained using FlowJo7.6.2 Data Analysis Software. The assessment was repeated three times in independent experiments blind to experimenter and flow cytometer analyst (Adan et al. 2017).

### Analysis of Mitochondrial Membrane Potential (ΔΨm) by Fluorescence Microscopy

ChLNs were left in a regular culture medium (RCm) for 4 days. Then, the cells (5 × 10^3^) were incubated with the passively diffusing and active mitochondria-accumulating dye deep red MitoTracker compound (20 nM, final concentration) for 20 min at RT in the dark (cat # M22426, Invitrogen, Soto-Mercado et al. 2020). Cells were then washed twice with PBS. MitoTracker fluorescence intensity was determined by analysis of fluorescence microscopy images (Lichtman and Conchello 2005). The assessment was repeated three times in independent experiments. The nuclei were stained with 0.5 µM Hoechst 33,342 staining compound. The assessment was repeated three times in independent experiments blind to the experimenter.

### Analysis of Mitochondrial Membrane Potential ($\Delta \mathrm{\Psi m}$) by Flow Cytometry

ChLNs were left in a regular culture medium (RCm) for 4 days. Then, the cells (1 × 10^4^) were incubated with the passively diffusing and active mitochondria-accumulating dye deep red MitoTracker compound (20 nM, final concentration) for 20 min at RT in the dark (cat # M22426, Invitrogen, (Soto-Mercado et al. 2020). The cells were analyzed using an LSRFortessa (BD Biosciences). The experiment was performed three times in independent experiments, and 10,000 events were acquired for analysis. Quantitative data and figures were obtained using FlowJo 7.6.2 Data Analysis Software. The assessment was repeated three times in independent experiments blind to experimenter and flow cytometer analyst.

### Intracellular Calcium Imaging

Intracellular calcium (Ca^2+^) concentration changes evoked by cholinergic stimulation were assessed according to Sekiguchi-Tonosaki et al. 2009 and Pap et al. 2009, with minor modifications. For the measurement, the fluorescent dye Fluo-3 (Fluo-3 AM; Thermo Fisher Scientific, cat: F1242) was employed. The dye was dissolved in DMSO (1 mM) to form a stock solution. Before the experiments, the stock solution was diluted in neuronal buffer solution (NBS buffer in mM: 137 NaCl, 5 KCl, 2.5 CaCl_2_, 1 MgCl_2_, pH 7.3, and 22 glucose). The working concentration of the dye was 2 μM. The WT and PSEN1 E280A ChLNs were incubated for 30 min at 37 °C with the dye containing NBS and then washed five times. Intracellular Ca^2+^ transients were evoked by acetylcholine (1 mM final concentration) at 4 days post differentiation. The measurements were carried out using the 20 × objective of the microscope. Several regions of interest (ROIs) were defined in the visual field of the camera. One of the ROIs was cell free, and the fluorescence intensity measured here was considered background fluorescence (F_**b**ack**g**roung_). The time dependence of the fluorescence emission was acquired, and the fluorescence intensities (hence the Ca^2+^ levels) were represented by pseudo colors. To calculate the changes of the average Ca^2+^-related fluorescence intensities, the F_bg_ value was determined from the cell-free ROI, and then the resting fluorescence intensities (F_rest_) of the cell-containing ROIs were obtained as the average of the points recorded during a consecutive period of 10 s before the addition of acetylcholine. The peaks of the fluorescence transients were found by calculating the average of six consecutive points and identifying those points that gave the highest average value (F_max_). The amplitudes of the Ca^2+^-related fluorescence transients were expressed relative to the resting fluorescence (ΔF/F) and were calculated by the following formula: ΔF/F = (F_max_ − F_rest_)/(F_rest_ − F_bg_). For the calculation of the fluorescence intensities, ImageJ was used. The terms fluorescence intensity was used as an indirect indicator of intracellular Ca^2+^ concentration. The assessment was repeated three times in independent experiments blind to the experimenter.

### Photomicrography and Image Analysis

Light microscopy photographs were taken using a Zeiss Axio Vert.A1 coupled to AxioCam Cm1 microscope, and fluorescence microscopy photographs were taken using a Zeiss Axio Vert.A1 Fluorescence Microscope equipped with a Zeiss AxioCam Cm1. Fluorescence images were transformed into 8-bit images and the background was subtracted (Zen 3.4 blue edition). Images were then analyzed by ImageJ software (http://imagej.nih.gov/ij/) using an in house-made macro. Briefly, the cellular measurement regions of interest (ROIs) were drawn around the nucleus (for the case of transcription factors and apoptosis effectors) or overall cells (for cytoplasmic probes), and the fluorescence intensity was subsequently determined by applying the same threshold for cells in the control and treatment conditions. Mean fluorescence intensity (MFI) was obtained by normalizing total fluorescence to the number of nuclei.

### Molecular Docking

We used the x-ray diffraction crystallography protein structure of $\upgamma$-secretase (protein data bank, PDB, code: 5FN2, (Bai et al. 2015) for molecular docking analysis. The blind molecular docking was performed with CB-Dock version 2 (Liu et al. 2022), a cavity detection-guided protein–ligand blind docking web server that uses Autodock Vina (version 1.1.2, Scripps Research Institute, La Jolla, USA). The Standard Delay Format (SDF) of the chemical structure of the tested compounds (rotenone, SCH 697466, MRK560, SCH 900229, LY-374973) were downloaded from PubChem. The molecular blind docking was performed by uploading the 3D structure PDB file of listed protein into the server with the SDF file of each compound. For analysis, we selected the docking poses with the strongest Vina score in the catalytical pocket. The generated PDB files of the molecular docking of each compound were visualized with the CB-Dock2 interphase and were compared against the experimentally validated X-ray structures of the interaction with reference compounds.

### Data Analysis

In this experimental design, two vials of MSCs were thawed (WT PSEN1 and PSEN1 E280A), cultured and the cell suspension was pipetted at a standardized cellular density of 2.6 × 10^4^ cells/cm^2^ into different wells of a 24-well plate. Cells (i.e., the biological and observational unit (Lazic et al. 2018) were randomized to wells by simple randomization (sampling without replacement method), and then wells (i.e., the experimental units) were randomized to treatments by a similar method. Experiments were performed on three independent occasions (n = 3) blind to the experimenter and/or flow cytometer analyst (Lazic et al. 2018). The data from the three repetitions i.e., independent experiments were averaged and a representative flow cytometry density or histogram plot from the three independent experiments was selected for illustrative purposes, whereas bar in quantification figures represent the mean ± SD and the three black dots show data point of each experimental repetition. Based on the assumption that the experimental unit (i.e., the well) data comply with the independence of observations, the dependent variable is normally distributed in each treatment group (Shapiro–Wilk test), and variances are homogeneous (Levene’s test), the statistical significance was determined by one-way analysis of variance (ANOVA) followed by Tukey’s post hoc comparison calculated with GraphPad Prism 5.0 software. Differences between groups were only deemed significant when a p-value of < 0.05 (*), < 0.001 (**) and < 0.001 (***). All data are illustrated as the mean ± S.D.

## Results

### Wild Type (WT) and PSEN 1 E280 Warthon Jelly’s Mesenchymal Stromal Cells (WJ MSCs)-Derived Cholinergic-Like Cells (ChLNs) Show Typical Markers of Cholinergic Lineage

**Fig. 1:**
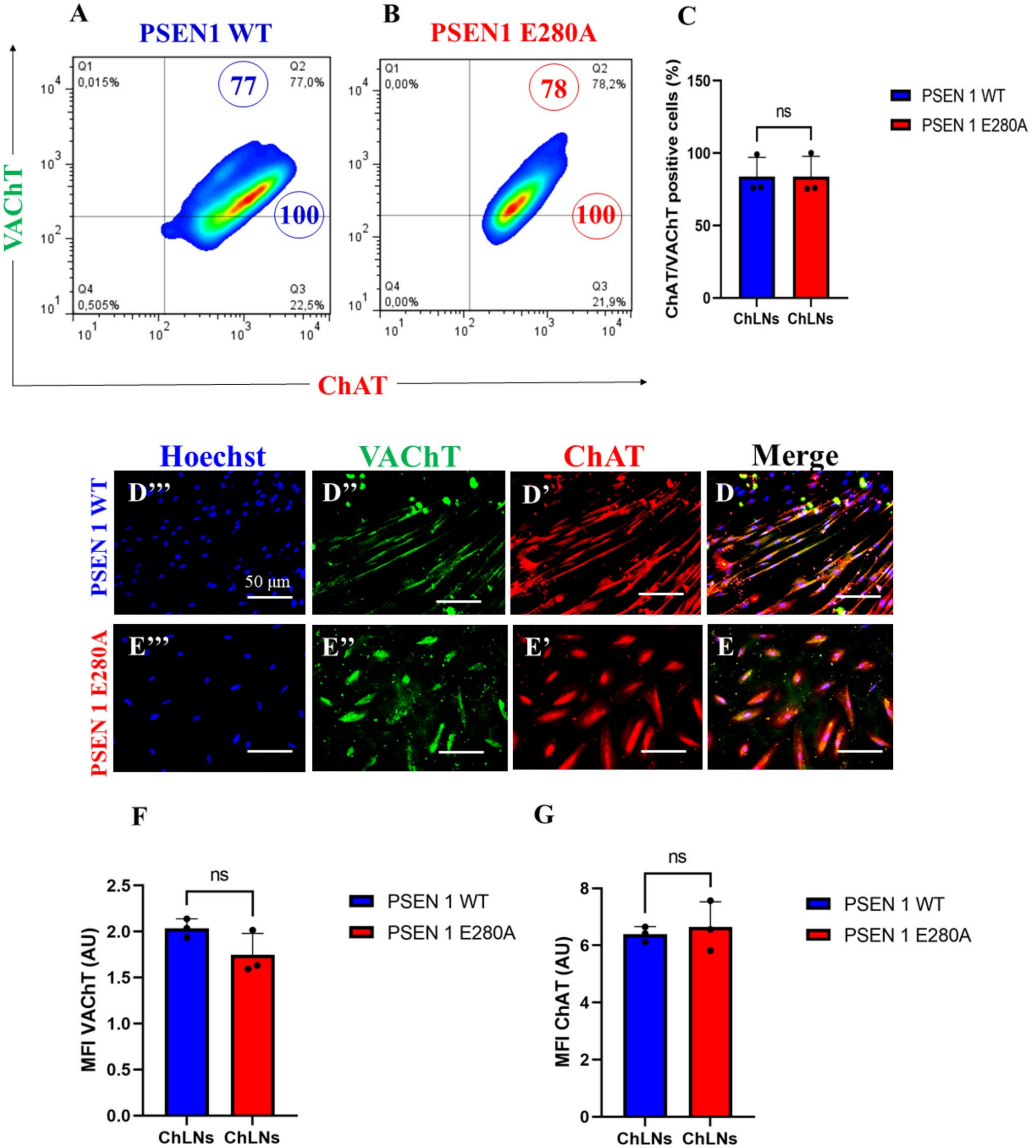
Wild type (WT) and PSEN 1 E280A Warthon Jelly’s Mesenchymal Stromal Cells (WJ MSCs)-derived Cholinergic-Like cells (ChLNs) show typical markers of cholinergic lineage. WT PSEN1 and PSEN1 E280A MenSCs Warthon Jelly’s Mesenchymal Stromal Cells (WJ MSCs) were cultured in a cholinergic differentiation medium as described in the *Materials and Methods* section for 7 days. Cells were double stained with primary antibodies against the cholinergic lineage proteins vacuole acetylcholine transporter (VAChT) and cholinergic acetylcholine transferase (ChAT). Representative flow cytometry density plots showing **A** VAChT + / ChAT + PSEN1 WT ChLNs, **B** VAChT + / ChAT + PSEN1 E280A ChLNs. **C** Percentage of VAChT- and ChAT-positive cells in WT PSEN1 and PSEN1 E280A ChLNs. Representative fluorescence microscopy photographs showing **D** merge image of **D’** ChAT, **D”** VAChT, and **D’’’** Hoechst WT PSEN1 ChLNs; **E** merge image of **E’** ChAT, **E”** VAChT, and **E’’’** Hoechst PSEN1 E280A ChLNs. Positive blue fluorescence reflects nuclei, positive red fluorescence reflects the presence of ChAT, positive green fluorescence reflects the presence of VAChT protein. **F** Mean fluorescence intensity (MFI) of VAChT. **G** Mean fluorescence intensity (MFI) of ChAT. The data are presented as the mean ± SD of three independent experiments (n = 3). The figures represent 1 out of 3 independent experiments. One-way ANOVA followed by Tukey’s test; n.s. = no statistically significance. Image magnification, 200×

We first wanted to confirm that the mutation PSEN 1 E280A did not alter the transdifferentiation of WJ-MSCs into ChLNs cultured in *Ch–N-Run* medium (Soto-Mercado et al. 2020; Mendivil-Perez et al. 2023). Effectively, WT and mutant WJ-MSCs transdifferentiated into ChLNs in *Cholinergic-N-Run* yielding 75% cholinergic markers ChAT/ VAChT according to flow cytometry analysis (Fig. 1A-C). The cholinergic lineage markers were also detected by fluorescent microscopy (Fig. 1D–G). For comparative and validation purposes, we included the PSEN1 E280A ChLNs (Soto-Mercado et al. 2020; Mendivil-Perez et al. 2023).

### Rotenone (ROT) Provokes Loss of Mitochondrial Membrane Potential ($\Delta \mathrm{\Psi m}$) and Generation of Reactive Oxygen Species (ROS)

**Fig. 2:**
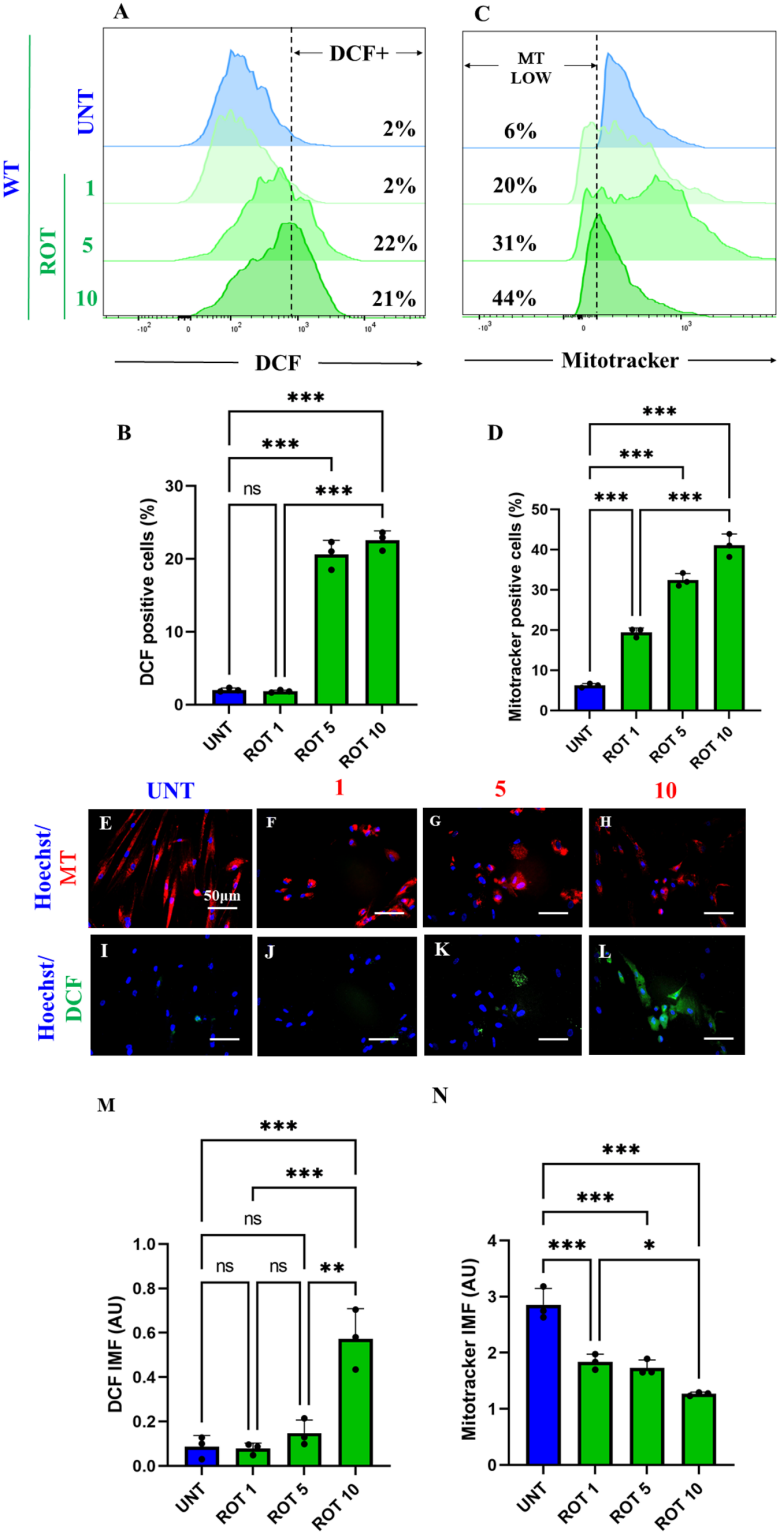
Rotenone (ROT) provokes loss of mitochondrial membrane potential ($\Delta \mathrm{\Psi m}$) and generation of reactive oxygen species (ROS). After 7 days of transdifferentiation, WT PSEN1 ChLNs were left untreated or treated with increasing concentrations of ROT (0, 1, 5, 10 $\upmu$M) in regular culture medium (RCm) for 24 h. **A** Representative flow cytometry histogram showing DCF-positive (DCF +). **B** Quantification of DCF-positive cells by flow cytometry. **C** Representative flow cytometry histogram showing ΔΨm. **D** Quantification of ΔΨm by flow cytometry. **E**–**H** Representative fluorescent microscopy showing WT PSEN 1 ChLNs treated whit ROT stained with MitoTracker™ Red. **I**–**L** Representative fluorescent microscopy showing WT PSEN1 ChLNs treated whit ROT stained with DCF. Positive blue fluorescence reflects nuclei, positive red fluorescence reflects $\Delta \mathrm{\Psi m}$, positive green fluorescence reflects the presence DCF + cells. **M** Mean fluorescence intensity (MFI) of DCF-positive cells **N** Mean fluorescence intensity (MFI) of ΔΨm. Data are expressed as mean ± SD; Statistically significant differences when *p < 0.05; ** p < 0.01; ***p < 0.001. The figures represent 1 out of 3 independent experiments. Image magnification, 200×

It is well-known that ROT induces ROS/H_2_O_2_ through inhibition of mitochondrial Complex I (Li et al. 2003; Read et al. 2021). Therefore, we determined the concentration of ROT at which generates the maximal percentage of ROS and mitochondrial damage in ChLNs. As shown in Fig. 2, ROT induces concentration-dependent loss of $\Delta \mathrm{\Psi m}$ (Fig. 2A, B) and generation of ROS up to 5 μM (Fig. 2C, D). Similar data were observed by fluorescent microscopy (Fig. 2E–N). Therefore, we selected ROT (10 $\upmu$M) for further experiments.

### Rotenone (ROT) Induces Phosphorylation of Leucine-Rich Repeated Kinase 2 (LRRK2) Concomitantly with Phosphorylation of $\mathrm{\alpha }$-Synuclein at Pathological Residue Ser^129^ in ChLNs

**Fig. 3:**
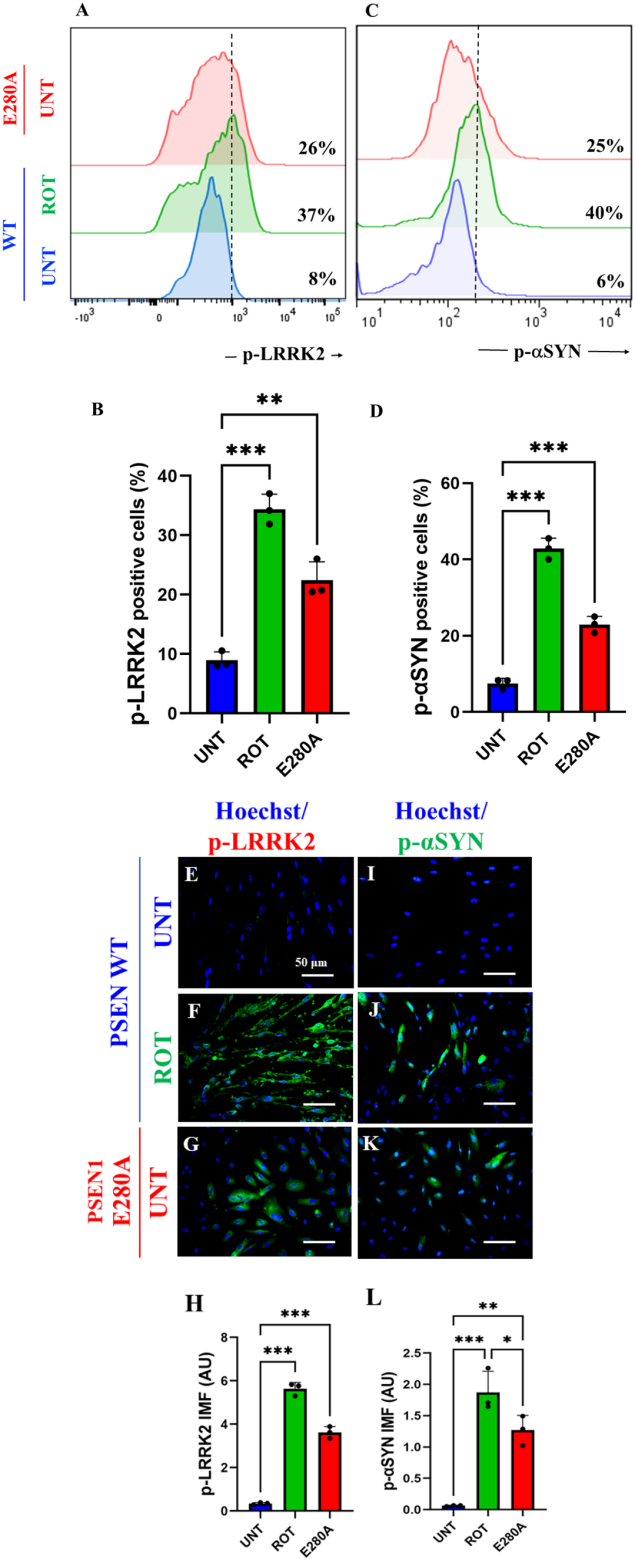
Rotenone (ROT) induces phosphorylation of leucine-rich repeated kinase 2 (LRRK2) concomitantly with phosphorylation of $\mathrm{\alpha }$-synuclein at pathological residue Ser^129^ in ChLNs. After 7 days of transdifferentiation, WT PSEN1 ChLNs were untreated or treated with ROT 10 $\upmu$M and PSEN1 E280A ChLNs were left in regular culture medium (RCm) for 24 h. **A** Representative flow cytometry histogram showing p-LRRK2. **B** Quantification of p-LRRK2-positive cells by flow cytometry, **C** Representative flow cytometry histogram showing p-αSYN. **D** Mean fluorescence intensity (MFI) of p-αSYN. **E**–**H** Representative fluorescence microscopy photographs showing **E** p-LRRK2 in untreated PSEN1 WT ChLNs or **F** treated with ROT. Representative fluorescence microscopy photographs showing **G** p-LRRK2-positive cells in PSEN1 E280A ChLNs. Positive blue fluorescence reflects nuclei, positive green fluorescence reflects the presence of p-LRRK2 protein. **H** Mean fluorescence intensity (MFI) of p-LRRK2. **I**–**K** Representative fluorescence microscopy photographs showing **I** p-αSYN in untreated PSEN1 WT ChLNs or **J** treated with ROT. Representative fluorescence microscopy photographs showing **K** p-αSYN -positive cells in PSEN1 E280A ChLNs. Positive blue fluorescence reflects nuclei, positive green fluorescence reflects the presence of p-αSYN protein. **L** Mean fluorescence intensity (MFI) of p-αSYN. Data are expressed as mean ± SD; Statistically significant differences when *p < 0.05; ** p < 0.01; ***p < 0.001. The figures represent 1 out of 3 independent experiments. Image magnification, 200×

Next, we determined whether ROT induces p-LRRK2 concomitantly with p-$\mathrm{ \alpha }$-Syn in ChLNs. To achieve this, ChLNs were exposed to ROT (10 $\upmu$M) for 24 h and then p-LRRK2 at residue Ser^935^ and p-$\mathrm{ \alpha }$-Syn at residue Ser^129^ were evaluated. Flow cytometry analysis reveals that ROT increased the phosphorylation of both LRRK2 by + 363% (Fig. 3A, B) and $\mathrm{\alpha }$-Syn by + 567% (Fig. 3C, D) compared to untreated ChLNs. Surprisingly, mutant ChLNs showed an important increased in p-LRRK2 (+ 225%), and p-$\mathrm{ \alpha }$-Syn (+ 317%) compared to untreated ChLNs (Fig. 3A–D). Similar data were obtained by fluorescent microscopy analysis (Fig. 3E–L).

### Rotenone (ROT) Induces Accumulation of iA$\upbeta$, Oxidized DJ-1 (DJ-1Cys^106^-SO_3_) and Phosphorylation of TAU at Pathological Residue Ser^202/^Thr^205^ in ChLNs

**Fig. 4:**
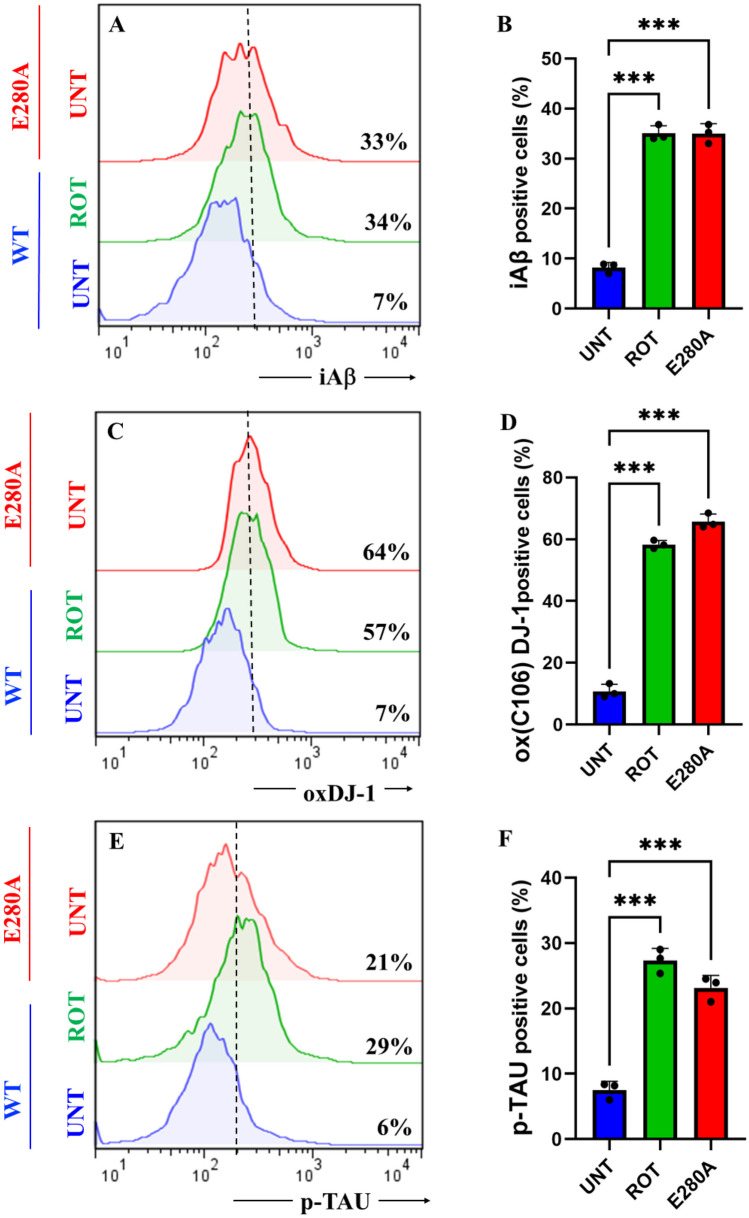

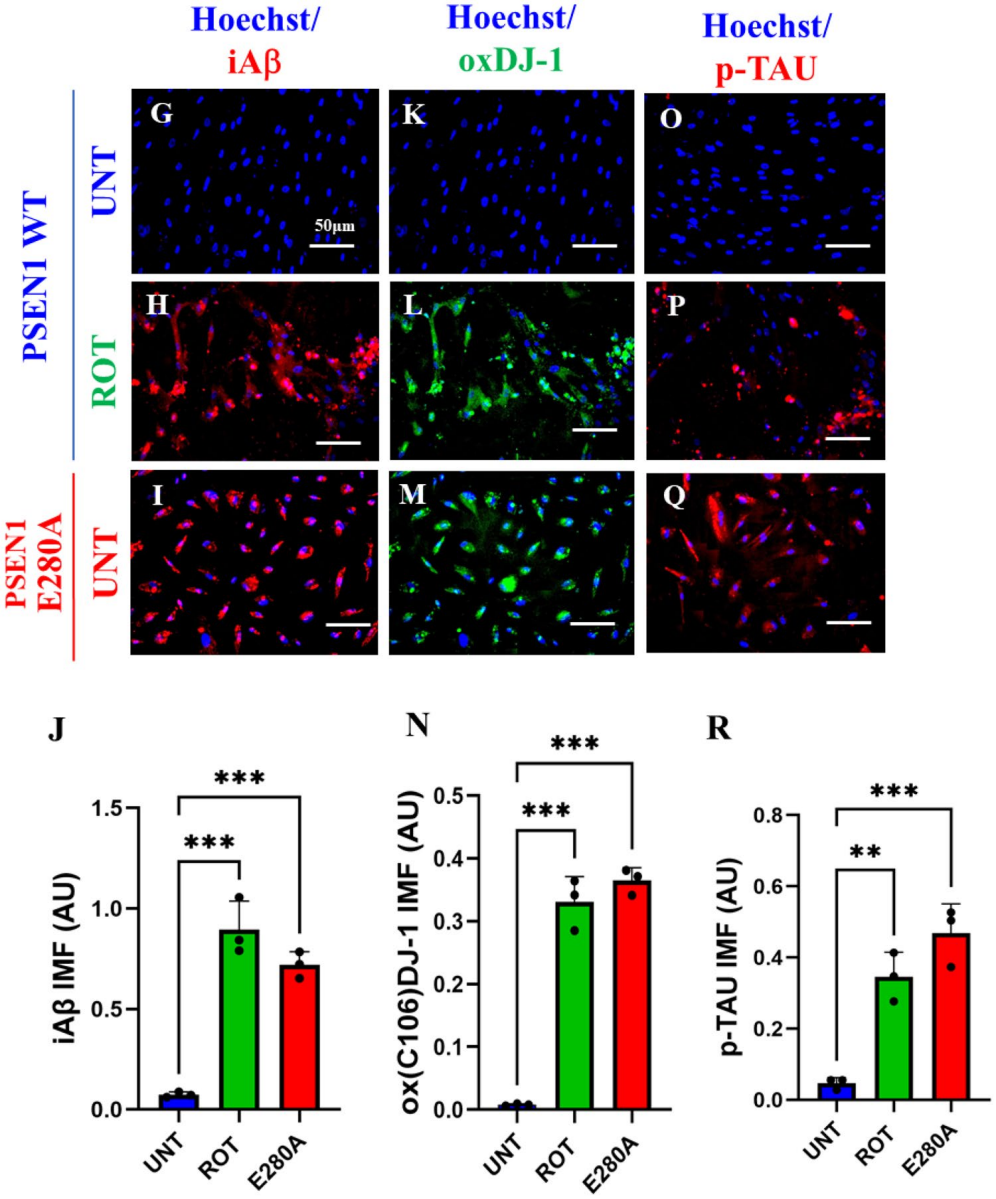
Rotenone (ROT) induces accumulation of iA$\upbeta$, oxidized DJ-1 (DJ-1Cys^106^-SO_3_) and phosphorylation of TAU at pathological residue Ser^202/^Thr^205^ in ChLNs. After 7 days of transdifferentiation, WT PSEN1 ChLNs were untreated or treated with ROT 10 μM and PSEN1 E280A ChLNs were left in regular culture medium (RCm) for 24 h. **A** Representative flow cytometry histogram showing iAβ. **B** Quantification of iAβ by flow cytometry, **C** Representative histogram showing oxDJ-1 by flow cytometry. **D** Mean fluorescence intensity (MFI) of oxDJ-1. **E** Representative histogram showing p-TAU by flow cytometry. **F** Mean fluorescence intensity (MFI) of p-TAU. **G**–**I** Representative fluorescence microscopy photographs showing **G** iAβ in untreated PSEN1 WT ChLNs or **H** treated with ROT. Representative fluorescence microscopy photographs showing **I** iAβ-positive cells in PSEN1 E280A ChLNs. Positive blue fluorescence reflects nuclei, positive red fluorescence reflects the presence of iAβ protein. **J** Mean fluorescence intensity (MFI) of iAβ. **K**–**M** Representative fluorescence microscopy photographs showing **K** Representative fluorescence microscopy photographs showing oxDJ-1 in untreated PSEN1 WT ChLNs or **L** treated with ROT. Representative fluorescence microscopy photographs showing **M** oxDJ-1-positive cells in PSEN1 E280A ChLNs. Positive blue fluorescence reflects nuclei, positive green fluorescence reflects the presence of oxDJ-1 protein. **N** Mean fluorescence intensity (MFI) of oxDJ-1. **O**–**R** Representative fluorescence microscopy photographs showing **O** p-TAU in untreated PSEN1 WT ChLNs or **P** treated with ROT. Representative fluorescence microscopy photographs showing **Q** p-TAU-positive cells in PSEN1 E280A ChLNs. Positive blue fluorescence reflects nuclei, positive red fluorescence reflects the presence of p-TAU protein. **R** Mean fluorescence intensity (MFI) of p-TAU. Data are expressed as mean ± SD; Statistically significant differences when *p < 0.05; ** p < 0.01; ***p < 0.001. The figures represent 1 out of 3 independent experiments. Image magnification, 200×

To characterize the neuronal expression of A$\upbeta$ peptides in ChLNs under ROT exposure, we chose an antibody that has previously been shown to be specific against A$\upbeta$_42_, namely the A$\upbeta$4 1E8 antibody (Wiltfang et al. 2001; Maler et al. 2007). Thus, ChLNs were exposed to ROT (10 $\upmu$M) for 24 h and stained with the anti-amyloid A$\upbeta$4 1E8. Figure 4 shows that ROT increased the accumulation of iA$\upbeta$ by + 386% (Fig. 4A, B), oxidized DJ-1 by + 714% (Fig. 4C, D), and p-TAU at residue Ser^202^/Thr^205^ by + 383% (Fig. 4E, F) compared to untreated ChLNs. As expected, mutant ChLNs increased accumulated iA$\upbeta$ by + 371%, DJ-1Cys^106^-SO_3_ by + 814%, and p-TAU by + 250% (Fig. 4A–F). Similar data was obtained by fluorescent microscopy (Fig. 4G–R).

### Rotenone (ROT) Increases the Phosphorylation of c-JUN at Residues Ser^63^/Ser^73^ and the Expression of TP53, PUMA and CC3 in ChLNs

**Fig. 5:**
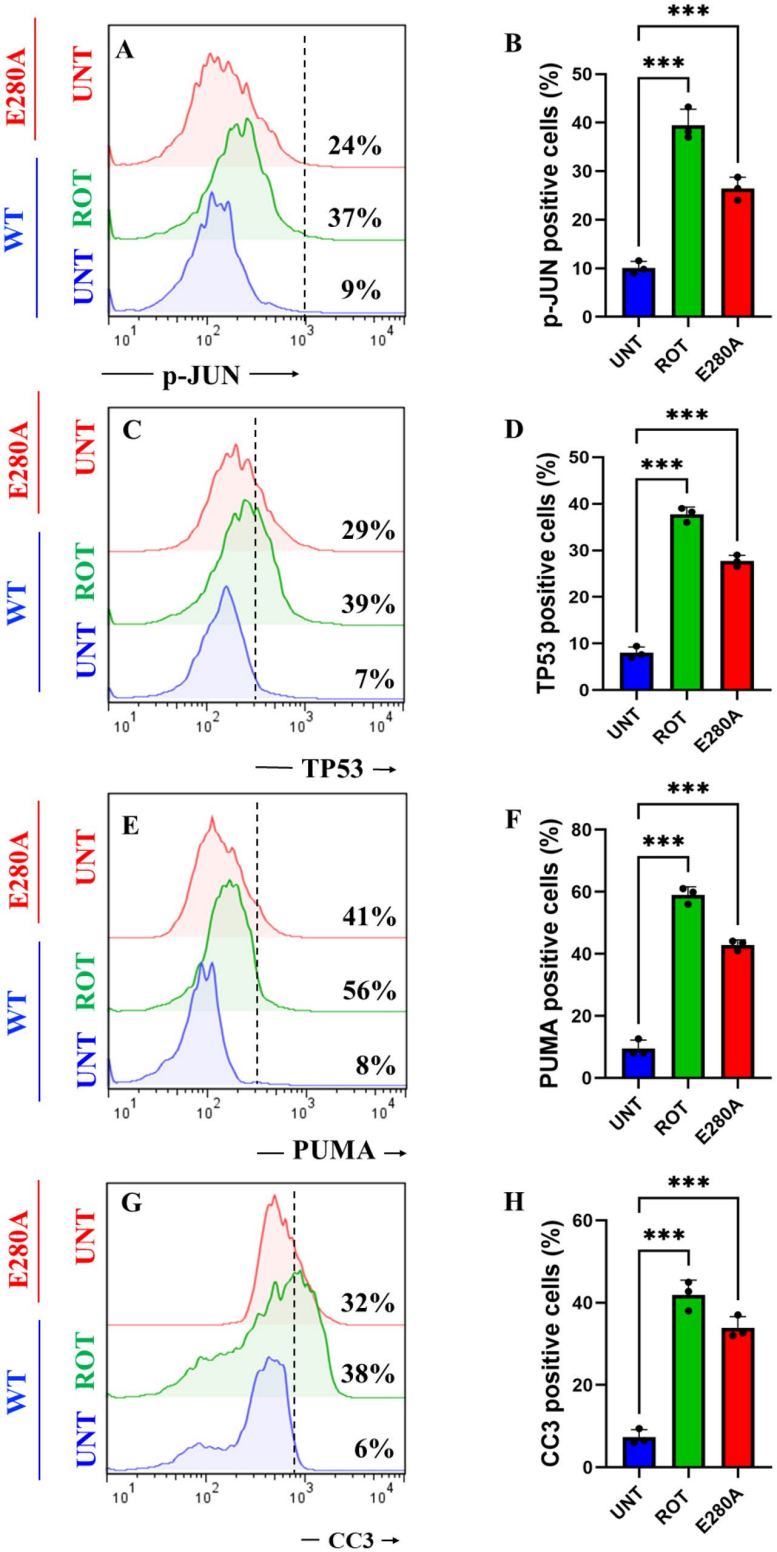

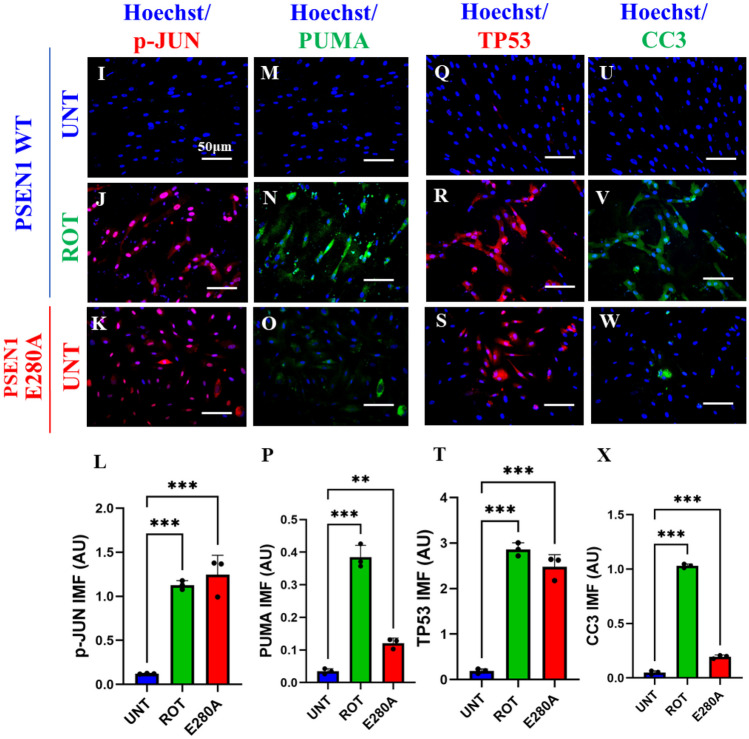
Rotenone (ROT) increases the phosphorylation of c-JUN at residues Ser^63^/Ser^73^ and the expression of TP53, PUMA and CC3 in ChLNs. After 7 days of transdifferentiation, WT PSEN1 ChLNs were untreated or treated with ROT 10 $\upmu$M and PSEN1 E280A ChLNs were left in regular culture medium (RCm) for 24 h. **A** Representative flow cytometry histogram showing p-JUN. **B** Mean fluorescence intensity (MFI) of p-JUN. **C** Representative flow cytometry histogram showing TP53. **D** Mean fluorescence intensity (MFI) of TP53-positive cells. **E** Representative flow cytometry histogram showing PUMA. **F** Mean fluorescence intensity (MFI) of PUMA-positive cells. **G** Representative flow cytometry histogram showing CC3. **H** Mean fluorescence intensity (MFI) of CC3-positive cells. **I**–**K** Representative fluorescence microscopy photographs showing **I** p-JUN in untreated PSEN1 WT ChLNs or **J** treated with ROT. Representative fluorescence microscopy photographs showing **K** p-JUN-positive cells in PSEN1 E280A ChLNs. Positive blue fluorescence reflects nuclei, positive red fluorescence reflects the presence of p-JUN protein. **L** Mean fluorescence intensity (MFI) of p-JUN. **M**–**O** Representative fluorescence microscopy photographs showing **M** PUMA in untreated PSEN1 WT ChLNs or **N** treated with ROT. Representative fluorescence microscopy photographs showing **O** PUMA-positive cells in PSEN1 E280A ChLNs. Positive blue fluorescence reflects nuclei, positive green fluorescence reflects the presence of PUMA protein. **P** Mean fluorescence intensity (MFI) of PUMA. **Q**–**T** Representative fluorescence microscopy photographs showing **Q** TP53 in untreated PSEN1 WT ChLNs or **R** treated with ROT. Representative fluorescence microscopy photographs showing **S** TP53-positive cells in PSEN1 E280A ChLNs. Positive blue fluorescence reflects nuclei, positive red fluorescence reflects the presence of TP53 protein. **T** Mean fluorescence intensity (MFI) of TP53. **U**–**W** Representative fluorescence microscopy photographs showing **U** CC3 in untreated PSEN1 WT ChLNs or **V** treated with ROT. Representative fluorescence microscopy photographs showing **W** CC3-positive cells in PSEN1 E280A ChLNs. Positive blue fluorescence reflects nuclei, positive green fluorescence reflects the presence of CC3 protein. **X** Mean fluorescence intensity (MFI) of CC3. Data are expressed as mean ± SD; Statistically significant differences when *p < 0.05; ** p < 0.01; ***p < 0.001. The figures represent 1 out of 3 independent experiments. Image magnification, 200×

**Fig. 6:**
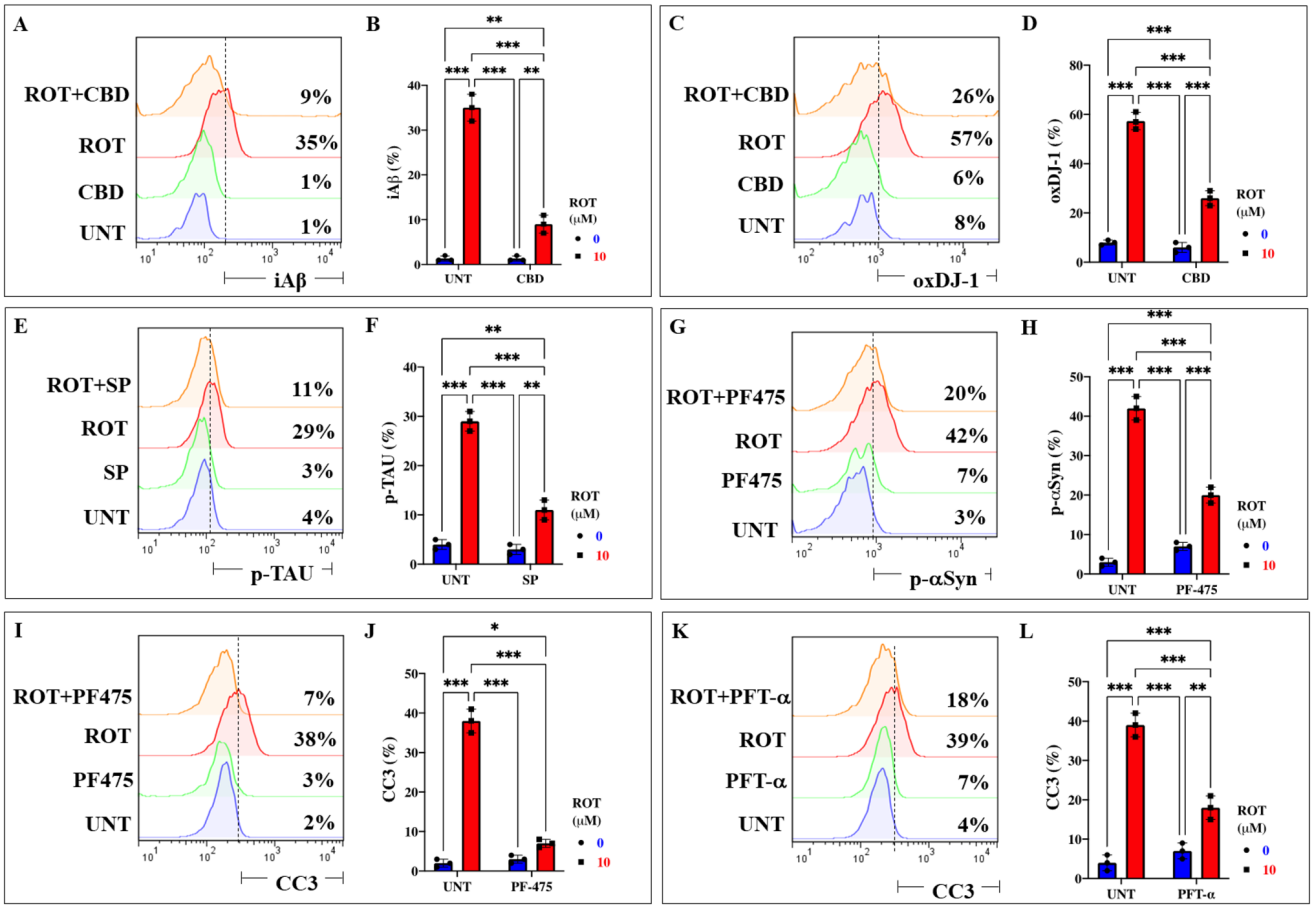
Cannabidiol (CBD), SP600125 (SP), pifithrin-$\mathrm{ \alpha }$ (PFT), and PF-06447475 (PF475) block ROT-induced accumulation of iA$\upbeta$, oxidized DJ-1, p-TAU, p-$\mathrm{ \alpha }$-Syn^129^, and CC3, respectively, in ChLNs. After 7 days of transdifferentiation, WT PSEN1 ChLNs were untreated or treated with ROT, with CBD (10 $\upmu$M), SP (1 $\upmu$M), and PF475 (1 $\upmu$M) in regular culture medium (RCm) for 24 h. **A** Representative flow cytometry histogram showing iA$\upbeta$ in untreated WT PSEN1 or treated untreated with ROT only, CBD only, or with ROT and CBD. **B** Percentage of iA$\upbeta$-positive cells by flow cytometry. **C** Representative flow cytometry histogram showing oxDJ-1 in untreated PSEN1 WT or treated with ROT only, CBD only, or with ROT and CBD. **D** Percentage of oxDJ-1-positive cells by flow cytometry. **E** Representative flow cytometry histogram showing p-TAU in untreated PSEN1 WT or treated with ROT only, SP only, or with ROT and SP. **F** Percentage of p-TAU-positive cells by flow cytometry. **G** Representative flow cytometry histogram showing p-$\mathrm{ \alpha }$-Syn in untreated PSEN1 WT or treated with ROT only, PF475 only, or with ROT and PF475. **H** Percentage of p-$\mathrm{ \alpha }$-Syn -positive cells by flow cytometry. **I** Representative flow cytometry histogram showing CC3 in untreated PSEN1 WT or treated with ROT only, PF475 only, or with ROT and PF475. **J** Percentage of CC3-positive cells by flow cytometry. **K** Representative flow cytometry histogram showing CC3 in untreated PSEN1 WT or treated with ROT only, PFT-$\mathrm{ \alpha }$ only, or with ROT and PFT-$\mathrm{ \alpha }$. **L** Percentage of p-$\mathrm{ \alpha }$-Syn -positive cells by flow cytometry. Data are expressed as mean ± SD; Statistically significant differences when *p < 0.05; ** p < 0.01; ***p < 0.001

We determined whether ROT induces apoptotic markers p-JUN, TP53, PUMA, and CC3 in ChLNs (Mendivil-Perez et al. 2016). As shown in Fig. 6, ROT induced an important increase in the phosphorylation of p-JUN, expression TP53, PUMA, and CC3 by + 311% (Fig. 5A, B), + 457% (Fig. 5C, D), + 600% (Fig. 5E, F), and + 533% (Fig. 5G, H), respectively, in ChLNs. Compared to untreated WT, PSEN1 E280A also endogenously expressed p-JUN, TP53, PUMA, and CC3 by + 167%, + 314%, + 412%, and + 433%, respectively (Fig. 5A–H). Similar data were obtained by fluorescent microscopy (Fig. 5I–X).

### Cannabidiol (CBD), SP600125 (SP), Pifithrin-$\mathrm{\alpha }$ (PFT) and PF-06447475 (PF475) Block ROT-Induced Accumulation of iA$\upbeta$, Oxidized DJ-1, p-TAU Ser^202^/Thr^205^, p-$\mathrm{ \alpha }$-Syn^129^, and CC3, Respectively, in ChLNs

**Fig. 7:**
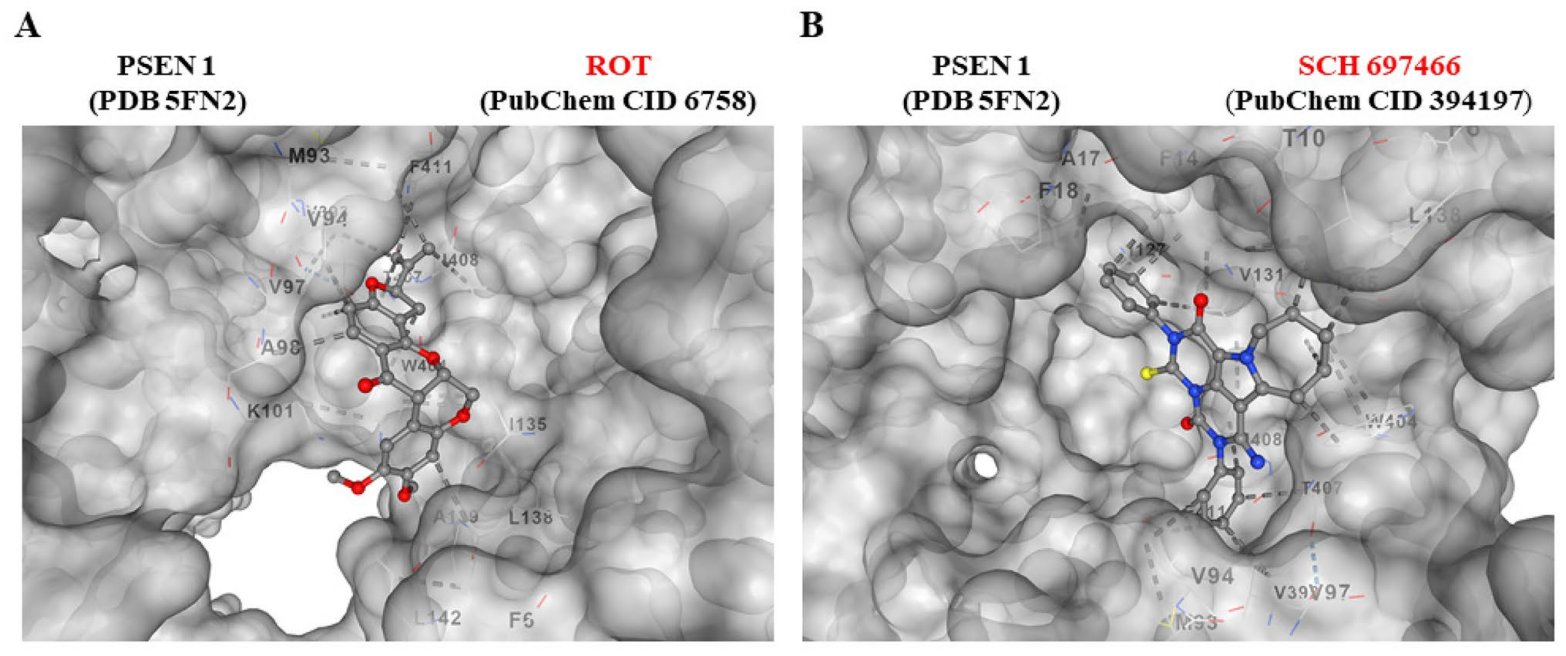
Rotenone binds to PSEN1/$\upgamma$-secretase. **A** Representative CB-Dock2 3D images showing the molecular docking of PSEN1 (PDB: 5FN2) with rotenone compound. **B** Molecular docking of PSEN1 (PDB: 5FN2) with inhibitor SCH 697466 compound

The above observations compelled us to evaluate whether the use of antioxidant or inhibitor compounds of critical molecules (e.g., iA$\upbeta$, DJ-1, TAU, $\mathrm{\alpha }$-Syn, CC3) diminish or augment ROT-induced signaling. To achieve this, ChLNs exposed to ROT alone or in combination with anti-amyloidogenic and antioxidant cannabidiol (CBD, 10 $\upmu$M), JNK inhibitor SP600125 (SP, 1 $\upmu$M), TP53 inhibitor pifithrin-$\mathrm{ \alpha }$ (PFT-$\mathrm{ \alpha }$, 50 nM), and LRRK2 inhibitor PF-06447475 (PF475, 1 $\upmu$M) were evaluated for their effect on the accumulated iA$\upbeta$, oxDJ-1, p-$\mathrm{ \alpha }$-Syn, p-TAU, and CC3, respectively. Figure 6 shows that CBD reduced the accumulation of iA$\upbeta$ by -74% (Fig. 6A, B) and DJ-1Cys^106^-SO_3_ by -54% (Fig. 6C, D); SP diminished p-TAU at Ser^202^/Thr^205^ by -62% (Fig. 6E, F), PF475 abridged p-$\mathrm{ \alpha }$-Syn at Ser^129^ by -52% (Fig. 6G, H), and blunted CC3 by -82% (Fig. 6I, J). Last, PFT-$\mathrm{ \alpha }$ blocked CC3 by -54% (Fig. 6K, L). In addition, we assessed theoretically whether ROT binds to PSEN 1/ $\upgamma$ -secretase. For comparative purposes, we used well-known pharmacological inhibitors of PSEN 1/ $\upgamma$ -secretase complex e.g., SCH 697466, MRK560, SCH 900229, and GSI LY-374973 (Lee et al. 2011; Wu et al. 2012; Hyde et al. 2013; Sogorb-Esteve et al. 2018; Serneels et al. 2023). Molecular in silico docking analysis reveals that ROT (PubChem CID 6758) binds to catalytic pocket of PSEN1 (RSCB protein data bank e.g., 5FN2, (Bai et al. 2015)) with a binding affinity of -8.0 (kcal / mol) Vina score (Fig. 7A and Table 1) compare to the high binding affinity displayed by the $\upgamma$ -secretase inhibitor e.g., SCH 697466 (-9.2 Vina Score, Fig. 7B and Table 1; (Hyde et al. 2013). Interestingly, other binding affinities for PSEN 1 inhibitors e.g., MRK560 (Lee et al. 2011), SCH 900229 (Wu et al. 2012), and GSI LY-374973 (Sogorb-Esteve et al. 2018) were near to the binding Vina Score of ROT (Table 1).

**Table 1:** In silico molecular docking analysis of ROT, inhibitor agents, and PSEN 1/ $\upgamma$ secretase

**Submitted Protein**^a^	**Submitted** **Ligand**^b^	**Vina Score**^c^	**Cavity Volume** **(A**^**3**^**)**	**Center** **(x, y, z)**	**Docking** **size** **(x, y, z)**	**Contact** **residue**
**PSEN 1/** $\upgamma$** secretase** (**5FN2**)	**ROT** (PubChem CID 6758)	**-8.0**	**4571**	**128, 120, 115**	**35, 35, 35**	**Pocket C1** **Chain B:** MET93 VAL94 VAL97 ALA98 LYS101 VAL393 TRP404 THR407 ILE408 PHE411 **Chain C:** PHE6 ILE135 LEU138 ALA139 LEU142
**PSEN 1/** $\upgamma$** secretase** (**5FN2**)	**SCH 697466** (PubChem CID 394197)	**-9.2**	**4571**	**128, 120, 115**	**35, 35, 35**	**Pocket C1** **Chain B**: MET93 VAL94 VAL97 VAL393 TRP404 THR407 ILE408 PHE411 **Chain C**: **PHE6** THR10 PHE14 ALA17 PHE18 ILE127 VAL131 ILE135 LEU138
**PSEN 1/** $\upgamma$** secretase** (**5FN2**)	**MRK560** (PubChem CID 11577204)	**-8.4**	**4571**	**128, 120, 115**	**35, 35, 35**	**Pocket C1** **Chain B:** THR90 MET93 VAL94 VAL97 VAL393 TRP404 THR407 ILE408 PHE411 VAL412 LEU415 **Chain C:** PHE14 ALA17 PHE18 PHE21 LEU35 VAL36 ILE127 ILE128 VAL131
**PSEN 1/** $\upgamma$** secretase** (**5FN2**)	**SCH 900229** (PubChem CID 25164607)	**-8.3**	**4571**	**128, 120, 115**	**35, 35, 35**	**Pocket C1** **Chain B:** VAL97 VAL393 TRP404 THR407 ILE408 PHE411 VAL412 LEU415 **Chain C:** PHE14 ALA17 PHE18 LEU35 VAL36 ALA39 ILE127 ILE128 VAL131 ILE135
**PSEN 1/** $\upgamma$** secretase** (**5FN2**)	**LY-374973** (PubChem CID 5311272)	**-8.3**	**4571**	**128, 120, 115**	**35, 35, 35**	**Pocket C1** **Chain B:** ILE143 MET146 THR147 LEU150 LEU166 SER169 SER170 LEU173 ILE229 MET233 ILE253 THR256 ASP257 ALA260 LYS265 LEU282 PHE283 LEU383 GLY384 ASP385 ILE387 PHE388
$\mathrm{\alpha }$** 7nAChR** **(7KOQ)**	**ROT** (PubChem CID 6758)	**-7.6**	**6911**	**118, 140, 113**	**32, 34, 35**	**Pocket C2** **Chain A**: LYS45 ALA257 GLU258 MET260 ALA262 **Chain B**: ASP41 VAL42 GLU44 GLU172 TRP173 ARG205 TYR209 TYR210 LEU214 LEU255 VAL256 GLU258 ILE259
$\mathrm{\alpha }$** 7nAChR** **(7KOQ)**	**Methyllycaconitine** (PubChem CID 5288811)	**-7.2**	**6911**	**118, 140, 113**	**32, 34, 35**	**Pocket C2** **Chain A: LYS45** ASN46 ALA95 **Chain B:** MET40 ASP41 **VAL42** ASP43 **GLU44** LYS45 ASN46 VAL48 THR50 ILE122 LYS124 GLU258 ILE259

^a^According to RCSB Protein Data Base (https://www.rcsb.org/)

^b^According to PubChem database (https://pubchem.ncbi.nlm.nih.gov/)

^c^According to CB-dock2: An accurate protein-ligand bind cocking tool (https://cadd.labshare.cn/cb-dock2/php/index.php)

### Rotenone (ROT) Impairs ACh-induced Transient Ca^2+^ Influx in ChLNs

**Fig. 8:**
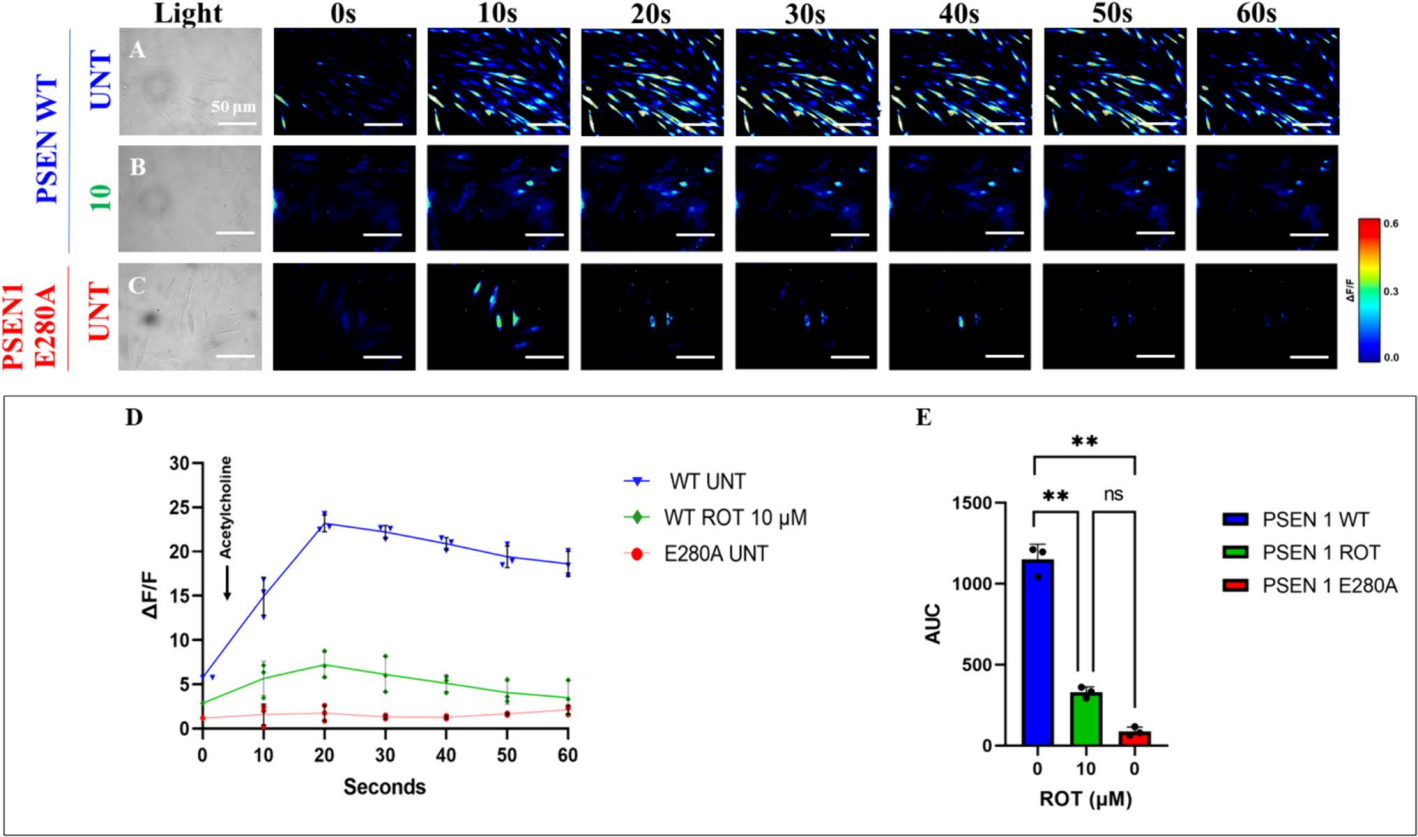
Rotenone (ROT) impairs acetylcholine (ACh)-induced transient Ca^2+^ influx in ChLNs. After 7 days of transdifferentiation, WT PSEN1 ChLNs were untreated or treated with ROT 10 $\upmu$M and PSEN1 E280A ChLNs were left in regular culture medium (RCm) for 24 h. **A**–**C** Time-lapse images (0, 10, 20, 30, 40, 50, and 60 s) of Ca^2+^ fluorescence in **A** untreated WT PSEN1 or **B** treated with ROT and **C** E280A ChLNs in response to ACh treatment. ACh was added to the culture at 0 s (arrow), and Ca^2+^ fluorescence of cells was monitored at the indicated times. The color contrast indicates fluorescence intensity: dark blue < light blue < green < yellow < red. **D** Normalized mean fluorescence signal (∆F/F) over time from cells indicating temporally elevated cytoplasmic Ca^2+^ in response to ACh treatment; **E** Calculated area under the curve (AUC). Data are expressed as mean ± SD; Statistically significant differences when ** p < 0.01. The figures represent 1 out of 3 independent experiments. Image magnification, 200×

We further evaluated whether ROT alters ChLNs response to ACh stimuli as an assessment of cholinergic neuronal Ca^2+^ responsiveness and functionality (Deutch and Roth 2014). To achieve this, ChLNs were left untreated or treated with ROT. Figure 8 shows that ACh stimulated a transient increase in intracellular Ca^2+^ in untreated ChLNs (Fig. 8A, ΔF/F = 23.5 ± 0.3, mean duration = 20 s; n = 20 ChLNs imaged, N = 3 dishes, Fig. 8D, E). In the presence of ROT, the Ca^2+^ influx was greatly reduced after ACh addition into ChLNs (Fig. 8B, D, ΔF/F = 6.00 ± 0.8, mean duration = 20 s; n = 20 ChLNs imaged, N = 3 dishes Fig. 8E). As expected, ACh did not affect intracellular dysfunctional Ca^2+^ influx in PSEN1 E280A ChLNs, used as control (Fig. 8C, ΔF/F = 0.50 ± 0.02, mean duration = 20 s; n = 20 ChLNs imaged, N = 3 dishes, Fig. 8D, E). We then theoretically inquired whether ROT might bind to $\mathrm{\alpha }$ 7 nicotinic cholinergic receptors ($\mathrm{\alpha }$7nChR), a cation permeable ligand-gated ion channels with a high permeability to Ca^2+^ (Noviello et al. 2021), which is implicated in AD (Singh et al. 2024). Molecular in silico docking analysis reveals that ROT binds to $\mathrm{\alpha }$ 7nChR with high affinity (-7.6 Vina score, Table 1) compared to e.g., methyllycaconitine (-7.2 Vina score, Table 1), a selective and potent antagonist of the α7nAChR at the $\mathrm{\alpha }$-bungarotoxin binding site (Ward et al. 1990).

## Discussion

In this study, we provide evidence that ROT induced the expression of the typical neuropathological hallmarks $\mathrm{\alpha }$-Syn, TAU, and iA$\upbeta$ in vitro cell model ChLNs. Interestingly, those pathological markers were found in PDD humans’ brains (Smith et al. 2019). Therefore, PDD is a neurological condition that recapitulates PD and AD (Goetz et al. 2009; Irwin et al. 2013; Jellinger 2023). Although previous studies have reported that ROT induces protein aggregation containing A$\upbeta$, p-α-Syn and hyperphosphorylated TAU in cultured cells of hippocampus, substantia nigra and locus coeruleus (Chaves et al. 2010, 2016), no further attempts were made to explain the mechanism by which ROT simultaneously triggers the coexistence of iA$\upbeta$, TAU, and $\mathrm{\alpha }$-Syn. Here, we show for the first time that ROT-induced coexistence of iA$\upbeta$, p-TAUSer^202^/Thr^205^, p-$\mathrm{ \alpha }$-SynSer^129^, and apoptosis signaling in ChLNs triggered by a cascade of molecular signaling, involving the oxidation of DJ-1 (DJ-Cys^106^-SO_3_), phosphorylation of LRRK2Ser^935^, activation of transcription factors c-JUN and TP53, expression of BH-3-only protein PUMA, loss of $\Delta \mathrm{\Psi m}$, and activation of CASP3. Furthermore, we found that LRRK2 kinase, c-Jun N-terminal kinase (JNK) signaling, ROT itself, and TP53/c-JUN/ PUMA are implicated in the phosphorylation of $\mathrm{\alpha }$-Syn, p-TAU, impairment of PSEN1/ ($\upgamma$-secretase complex), and apoptosis, respectively, in ChLNs. These findings might be of importance for the understanding of the mechanism(s) of neuronal cell death not only in PDD, but also in PD (Dong-Chen et al. 2023) and AD (Golde 2009).

Mounting evidence have demonstrated that ROT generates ROS/ H_2_O_2_ (Li et al. 2003) through inhibition of mitochondrial complex I (Read et al. 2021). In turn, H_2_O_2_ operates via oxidation of susceptible Cys-SH residue on proteins, thereby, mediating intracellular redox-sensitive signal transduction (Marinho et al. 2014; Di Marzo et al. 2018). In fact, H_2_O_2_ -induced apoptotic cell death through direct or indirect activation of several pro-death signaling molecules (Velez-Pardo and Jimenez-Del-Rio 2020), including kinases, transcription factors, and enzymes, among others. Here, we confirm that ROT induced concentration-dependent loss of $\Delta \mathrm{\Psi m}$, and generation of ROS/ H_2_O_2_ according to low-MitoTracker®-positive and DFC-positive ChLNs, respectively. Indeed, H_2_O_2_ not only induces depolarization of mitochondrial membrane (Takeyama et al. 2002) but also oxidizes the stress sensor protein DJ-1-Cys^106^-SH (*sulfhydryl* group) into the DJ-1-Cys^106^-SO_3_ (*sulfonic acid*). Actually, the Cys^106^-SH is the most sensitive cysteine residue in DJ-1 protein to H_2_O_2_-mediated oxidation (Kinumi et al. 2004). We found that ROT significantly increase the oxidized DJ-1 in ChLNs. Given that DJ-1 plays an important role in PD (Repici and Giorgini 2019), oxidized DJ-1 has been postulate as a possible biomarker of PD (Saito 2017). However, whether oxidized DJ-1 might be an additional pathologic marker associated with PDD merits further investigation. Nonetheless, ROS/ H_2_O_2_ disable DJ-1 from its capacity to modulate signaling pathways related with neuroprotective actions (Neves et al. 2022). Indeed, the most accepted function for DJ-1 is a neuronal protective role against OS (Biosa et al. 2017). Interestingly, the antioxidant CBD (Hacke et al. 2019) significantly reduced the oxDJ-1 in ChLNs exposed to ROT. Moreover, CBD has been demonstrated to inhibit the activation of CASP3 (Mendivil-Perez et al. 2023). Taken together, these observations suggest that rising of ROS/ H_2_O_2_ play a critical role in the early stages of the pathophysiology of the PDD. Therefore, antioxidant therapy should not only be beneficial for PDD but also for PD (Andrade et al. 2023).

How does ROT link $\mathrm{\alpha }$-Syn, iA$\upbeta$, and Tau in ChLNs as model of PDD? Our findings suggest that ROT triggers three alternative and complementary mechanisms, involving the putative interaction between ROT and PSEN1/ $\upgamma$ -secretase, ROT-induced activation of JNK, and phosphorylation of LRRK2 kinase, which eventually converge on p-$\mathrm{ \alpha }$-Syn, iA$\upbeta$, p-TAU, and apoptosis. Several observations support this. First, H_2_O_2_ might activate LRRK2 kinase activity by directly enhancing its autophosphorylation, e.g., at Tyr^1967^ (Kamikawaji et al. 2009), Ser^2032^, and Tyr^2035^ (West et al. 2007; Li et al. 2010), or indirectly, via phosphorylation of Ser^910^ and Ser^935^ via the inhibitor of nuclear factor-κB (IκB) kinase (IKK) complex (Dzamko et al. 2012). In line with this, we found a significant increase p-Ser^935^ LRRK2 concomitant with an important increase of p-Ser^129^ α-Syn in ChLNs exposed to ROT. These results suggest that, once active, the LRRK2 kinase phosphorylates α-Syn at Ser^129^ (Qing et al. 2009), which is the major component of pathological deposits in PD (Fujiwara et al. 2002; Du et al. 2021). Of note, the inhibitor LRRK2 kinase PF-06447475 almost completely abolished the p-Ser^129^-$\mathrm{ \alpha }$-Syn. Taken together, these results suggest that p-Ser^935^ LRRK2 is implicated in the phosphorylation of $\mathrm{\alpha }$-Syn at residue Ser^129^ in ChLNs treated with ROT (Qing et al. 2009). Second, H_2_O_2_ indirectly activates JNK kinase through activation of ASK-1 (Nadeau et al. 2009). In turn, JNK kinase phosphorylates TAU protein at Ser^202^/Thr^205^ (Reynolds et al. 2000), two amino acid residues implicated in TAU protein aggregation (Neddens et al. 2018). Interestingly, phosphorylation of TAU at residue Ser^208^, identified with antibody AT8 used in this work, also promotes aggregation and reveals neuropathologic diversity in Alzheimer’s disease and other tauopathies (Xia et al. 2020). Indeed, the combined phosphorylation at the Ser^202^/Thr^205^/Ser^208^ sites produces a Tau sample that readily forms fibers (Despres et al. 2017). Inhibition of the JNK signaling pathway might avoid TAU hyperphosphorylation and aggregation. Accordingly, we found that JNK inhibitor SP600125 significantly reduced p-TAU in ChLNs treated with ROT. Therefore, JNK is a potential therapeutic target for PDD, PD and AD (Hepp Rehfeldt et al. 2020; Usmani et al. 2021; Zhu et al. 2022). Nonetheless, LRRK2 may also contribute to TAU hyperphosphorylation and aggregation (Shanley et al. 2015; Hamm et al. 2015). Third, ROT induces high levels of accumulated iA$\upbeta$ in ChLNs. However, the molecular mechanism by which ROT induces iA$\upbeta$ is not yet fully established. A possible explanation is that ROT binds to PSEN 1/ $\upgamma$ -complex. Indeed, a molecular in silico docking analysis shows that ROT binds to a putative binding pocket in the PSEN1/ $\upgamma$ secretase with nearly similar binding Vina scores as those found with typical reference synthetic inhibitor PSEN1/ $\upgamma$ secretase MRK560, SCH 900229, and LY-374973 (Table 1). Although not yet experimentally confirmed, it is predicted that ROT might be able to affect dynamic conformational changes that control progressive catalysis by PSEN 1/ γ-secretase towards intracellular overproduction and accumulation of A$\upbeta$ (Svedružić et al. 2023). Indeed, the enzymatic activity of PSEN1/ $\upgamma$ -secretase is highly sensitive to structural changes induced by either PSEN1 mutations (Do et al. 2023) or $\upgamma$ secretase modulators GSM (Xia 2019). Interestingly, ROT displays mitochondrial off-target (e.g., binds to tubulin, Srivastava and Panda 2007) contributing to its toxic effects (Ren et al. 2005). Additionally, we found that CBD diminished accumulation of iA$\upbeta$ in ChLNs exposed to ROT. These observations suggest that CBD, in addition to operate as antioxidant, it also works as anti-amyloidogenic agent. Given that CBD possesses several pharmacological effects (Castillo-Arellano et al. 2023), this cannabinoid might be a potential therapeutic agent for the treatment of PDD (Ferreira-Junior et al. 2020; Zhang et al. 2022b; Mendivil-Perez et al. 2023). Finally, we confirm that ROT-induced apoptosis involving mitochondria depolarization and activation of CASP3 (Li et al. 2003) reflected as CC3 in ChLNs. Indeed, ROT increased the expression of TP53 and PUMA. Interestingly, both transcription factors TP53 and p–c-JUN transcribed PUMA (Nakano and Vousden 2001; Yu et al. 2001; Lu et al. 2014), a BH-3-only proapoptotic protein directly involved in mitochondrial depolarization and apoptosis (Roufayel et al. 2022; Czabotar and Garcia-Saez 2023), thereby contributing to the release of apoptogenic cytochrome C and activation of CASP3 (Dorstyn et al. 2018). Taken together, these observations imply that mitochondrial up-and downstream signaling are critical to trigger apoptosis in ChLNs. This last assumption is further supported by significant reduction of apoptosis in ChLNs when co-treated with inhibitor TP53 pifithrin-$\alpha$ or LRRK2 inhibitor PF-06447475 and ROT.

**Fig. 9:**
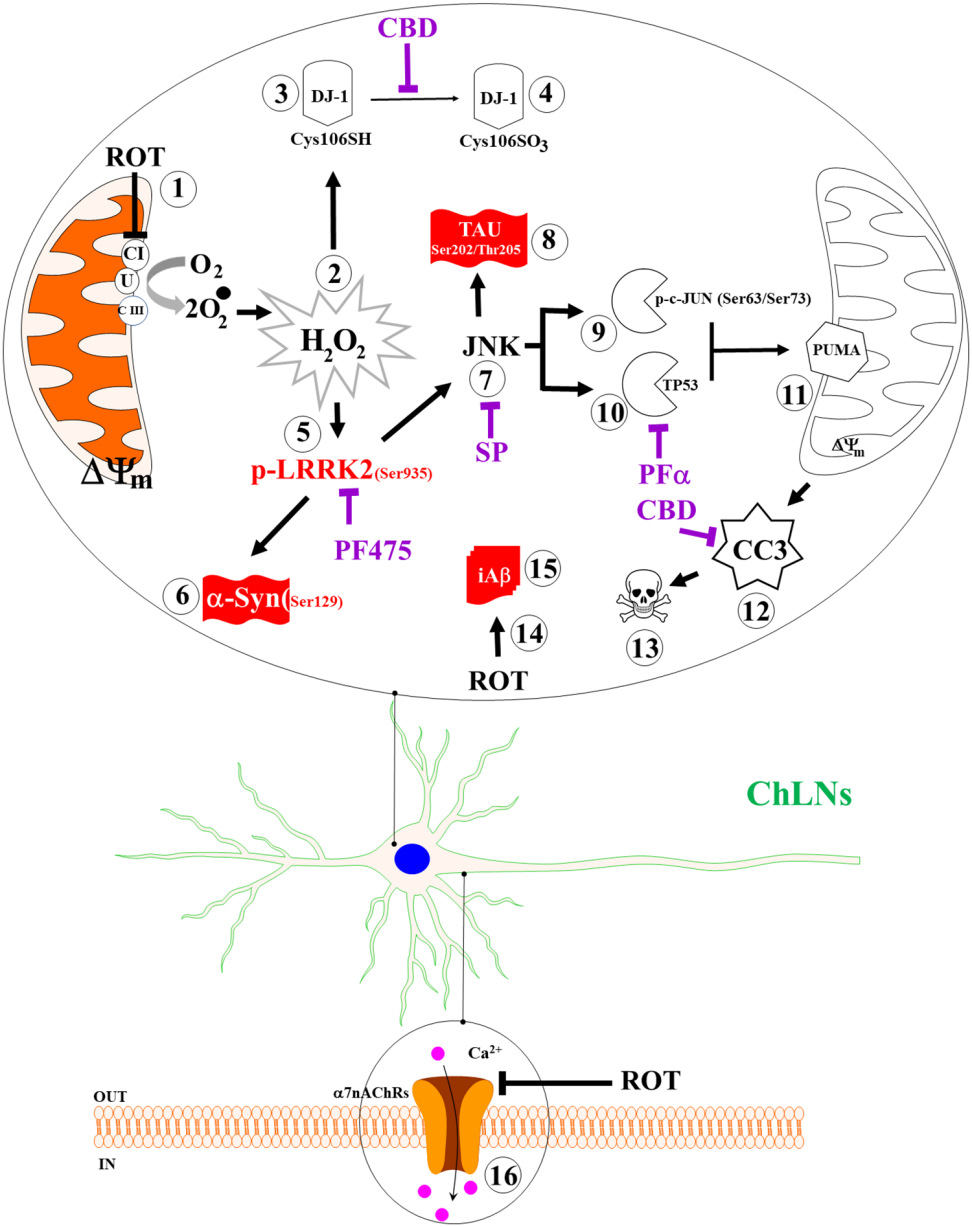
Schematic model of the effect of rotenone (ROT) on Cholinergic-Like neurons (ChLNs): A mechanistic explanation of the interaction between ROT, LRRK2 kinase, $\mathrm{\alpha }$-synuclein, TAU, and $\upbeta$. Rotenone (ROT) binds to the ubiquinone binding site of mitochondrial complex I preventing electron transfer through flavin mononucleotide (FMN) to coenzyme Q10 (1). Interruption of the electron transport chain concomitantly generates anion superoxide (O_2_^.−^) and hydrogen peroxide (H_2_O_2_, 2). This last compound is capable of directly oxidized DJ-1Cys^106^-SH (3) into DJ-1Cys^106^-SO_3_ (4), thereby disabling DJ-1 antioxidant signaling mechanisms. Alternatively, H_2_O_2_ activates LRRK 2 kinase (5) through activation of MEKK1/IKK or by autophosphorylation. Once LRRK 2 is phosphorylated at Ser^935^, the active kinase phosphorylates two major targets: (i) it phosphorylates $\mathrm{\alpha }$-Synuclein at Ser^129^ residue (6), and (ii) indirectly activates JNK pathway (7). This last kinase in turn phosphorylates protein TAU at Ser^202^/Tyr^205^ (8), activates via phosphorylation at Ser^63^/Ser^73^ pro-apoptotic c-JUN factor (9) and TP53 (10), which in turn transcribe PUMA (11), a BH-3-only Bcl-2 protein that induces mitochondria depolarization, resulting in cleaved caspase 3 (CC3, 12), which is responsible for chromatin condensation and DNA fragmentation (13), typical hallmarks of apoptosis. Additionally, ROT putatively binds to the PSEN1/$\upgamma$ -secretase (14), impairing the process of Amyloid $\upbeta$ Precursor Protein towards up-expression of internal accumulation of A$\upbeta$ (iA$\upbeta$, 15) and to the $\mathrm{\alpha }$7nAChRs (16), inducing Ca influx dysregulation in ChLNs. The coexistence of p-Ser^129^ $\mathrm{\alpha }$-Syn (6), p-Ser^202^/Thr^205^ TAU (8), and iA$\upbeta$ (15) constitute the pathological hallmark of PDD. Notably, selective inhibition of LRRK2, JNK, and TP 53 with PF475 (PF), SP600125 (SP), pifithrin-$\mathrm{ \alpha }$(PFT), and cannabidiol (CBD) blunted the appearance of p-$\mathrm{ \alpha }$-Syn (6), p-TAU (8), CC3 (12), condensed/ fragmented nuclei (13), and iA$\upbeta$ (15)

Additionally, ROT almost abolishes the ACh-induced transient intracellular Ca^2+^ flux in ChLNs. This suggests that ROT, similar to extracellular (e) A$\upbeta$ (Wang et al. 2000), somehow blocks the binding of ACh to ligand-gated Ca^2+^ ion channels (Uteshev 2012; Brown 2019). Moreover, docking analysis support the view that ROT can bind to nAChRs, e.g., $\mathrm{\alpha }$7nAChR with high affinity (Table 1). These observations might explain why ROT impairs learning and memory in mice (Guo et al. 2022). However, further investigation is needed to clarify these issues. We conclude that ROT-induced apoptosis, co-existence of p-$\mathrm{ \alpha }$-Syn, iA$\upbeta$, and p-TAU and neuronal Ca^2+^ influx dysfunction in ChLNs through OS-dependent and -independent mechanisms (Fig. 9).

For comparative purposes, we used PSEN 1 E280A (Soto-Mercado et al. 2020). Effectively, mutant ChLNs reproduced the pathological markers of Alzheimer’s disease, involving iA$\upbeta$, p-TAU, and oxDJ-1. Additionally, mutant ChLNs showed loss of $\Delta \mathrm{\Psi m}$, up regulation of p–c-JUN, TP53, PUMA, and CC3, leading to apoptosis and eA$\upbeta$-induced Ca^2+^ influx impairments (Soto-Mercado et al. 2020). Therefore, ROT and iA$\upbeta$ seem to be mechanistically homologous. Similar to ROT (this work), iA$\upbeta$ disrupts mitochondria bioenergetics (Sinclair et al. 2021), thereby generating ROS/H_2_O_2_, activates ASK-1 (Kadowaki et al. 2005), JNK/c-JUN axis, and p-TAU (Solas et al. 2023), and induces apoptosis dependent on CASP3 (Eimer and Vassar 2013). However, in contrast to ROT, A$\upbeta$ selectively inhibit Complex IV (Canevari et al. 1999). Surprisingly, we found for the first time an important endogenously increase in p-Ser^935^ LRRK 2 and p-Ser^129^ $\mathrm{\alpha }$-Syn in PSEN 1 E280A ChLNs compared to wild type ChLNs. Given that LRRK2 is an indispensable pro-apoptotic kinase (Quintero-Espinosa et al. 2017, 2023; Perez-Abshana et al. 2023), these results indicate that LRRK2 may play a major role in both DAergic and ChLNs demise. Furthermore, from a neuropathologic perspective, our findings might explain why some brains from demented patients show a mixed pathology of dementia with Lewy bodies (DLB) and AD (Kantarci et al. 2020). We report for the first time that PSEN 1 E280A ChLNs display the typical p-Ser^129^ $\mathrm{\alpha }$-Syn, a pathological feature found in PD, DLB, and PDD. However, whether p-Ser^129^ $\mathrm{\alpha }$-Syn and p-Ser^935^ LRRK2 are positive markers in human brains from FAD PSEN 1 E280A patients need further investigation.

Here, we show that ChLNs derived from WJ-MSCs treated with ROT might be an excellent system to model PDD. Our findings have one major implication for PD and PDD. It is known that the most significant nonmotor symptom in PD is progressive cognitive impairment (Aarsland et al. 2021), and PDD may affect 80% of PD patients long-term (Russell et al. 2014). Unfortunately, this recognition has not translated into significant treatment advances. One possible explanation for this drawback is the lack of proper in vitro or in vivo models. Here, we provide an in vitro model that accounts for the molecular mechanism by which ROT-induced cell death occurs in ChLNs, the major neuronal group involved in early AD and late PD. Furthermore, we found that LRRK2 is a key master kinase that links both PD through p-Ser^129^ $\mathrm{\alpha }$-Syn and FAD through apoptotic cell death signaling. This adds another layer of molecular complexity to the understanding of cell demise in both PD (Michel et al. 2016; Quintero-Espinosa et al. 2017, 2023; Velez-Pardo and Jimenez-Del-Rio 2020; Perez-Abshana et al. 2023) and FAD (Soto-Mercado et al. 2020; Brokaw et al. 2020). However, provided that LRRK2 is a druggable target (Thakur et al. 2022), it should be implemented in clinical trials including not only PDD but also PD as well as FAD patients. Our model system offers an understanding of the mechanisms of neurodegeneration in PD and AD. Nonetheless, our model has limitations. Although the data suggested that ROT impairs PSEN1 /$\upgamma$ -secretase towards increased accumulation of A$\upbeta$, there is no reasonable explanation for the endogenous production of iA$\upbeta$ in PDD brains. It is worth mentioning that a reformulation of the amyloid cascade hypothesis (ACH), now known as ACH 2.0, provides enlightenment on this issue (Volloch and Rits-Volloch 2023). Accordingly, it is proposed that AD is triggered by a first benign stage, wherein an APP-derived iA$\upbeta$ accumulated to sufficient levels in both sporadic (SAD) and FAD, and is driven by a second deleterious stage, wherein iA$\upbeta$ generated dependently of APP in FAD and independently of APP in SAD induced mitochondrial dysfunction and apoptosis (Volloch and Rits-Volloch 2023). Therefore, the role of iA$\upbeta$ and $\mathrm{\alpha }$-Syn pathology in the development of cognitive deficits in PD suggests that emerging disease-modifying therapies for AD may be beneficial for PDD patients.

## Conclusion

We provide an in vitro model recreating the neuropathology of PDD induced by ROT in ChLNs. Indeed, ROT induces p-$\mathrm{ \alpha }$-Syn, A$\upbeta$, p-Tau, and cell death in ChLNs. Furthermore, we identify the LRRK2 as master kinase that link both PD and AD via apoptotic cell death signaling. The “ChLNs plus ROT” approach provides an excellent platform to test for potential therapeutic strategies against PDD. Our data suggest that ROT induces a neuropathologic phenotype in ChLNs similar to that caused by the mutation PSEN1 E280A.

### Supplementary Information

Below is the link to the electronic supplementary material.

Supplementary file1 (PDF 1412 KB)

## Data Availability

No datasets were generated or analysed during the current study.

## Declarations

### Authors’ Information

D. G.-B. is a magister student of the Basic Biomedical Sciences Academic Corporation program at the Universidad de Antioquia (UdeA) funded by MinCiencias-Colombia. M.M.-P., C.V.-P. and M.J-R. are associated and senior professors, respectively, at the University of Antioquia.
