# Supplementary material for: Rotenone Induces a Neuropathological Phenotype in Cholinergic-like Neurons Resembling Parkinson’s Disease Dementia (PDD)
Source: Neurotox Res. 2024 Jun 6;42(3):28. doi: 10.1007/s12640-024-00705-3 (PMC11156752; doi:10.1007/s12640-024-00705-3)
Supplement: Supplementary file 1 — Supplementary file1 (PDF 1412 KB) [file 12640_2024_705_MOESM1_ESM.pdf]

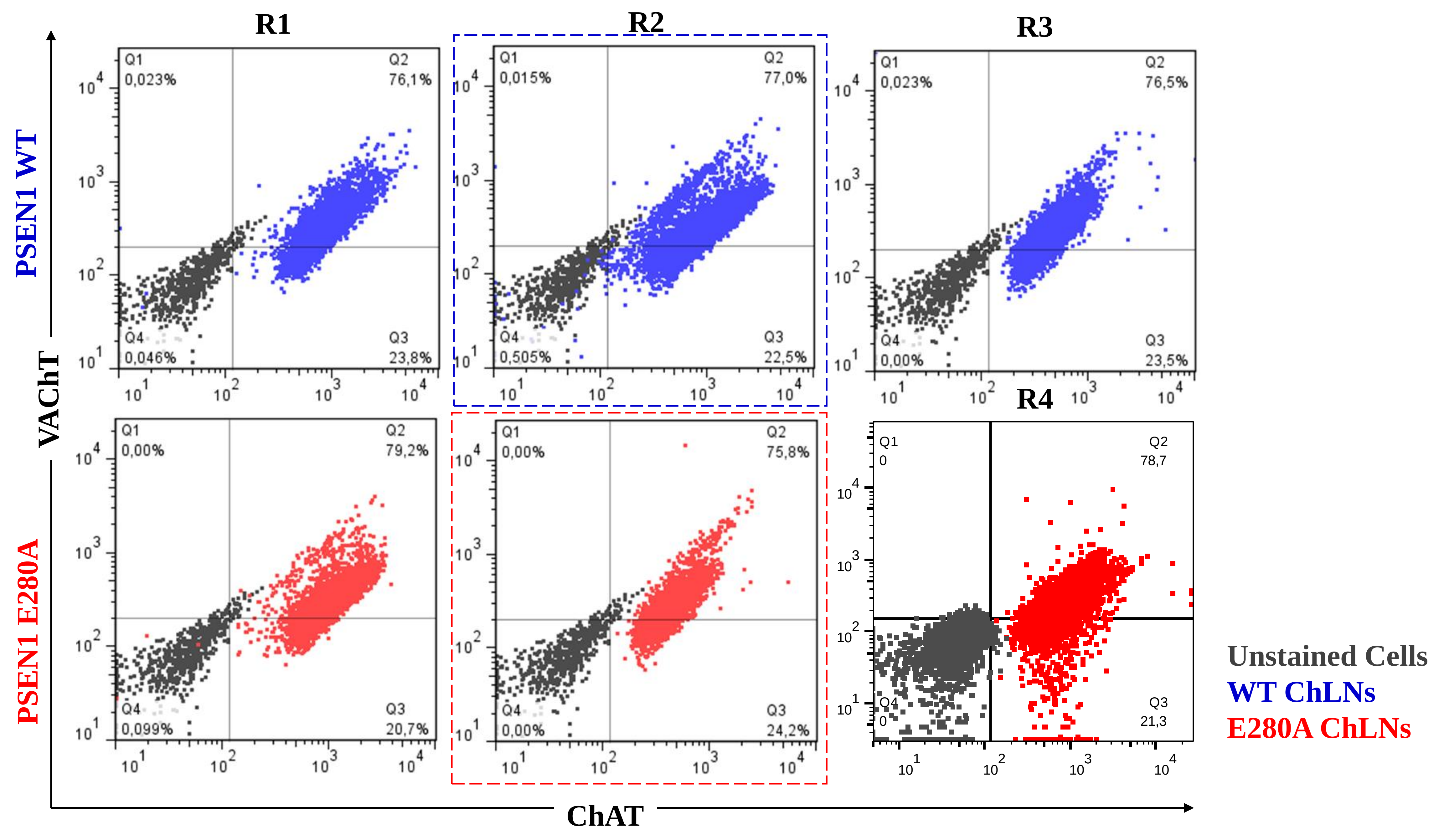

R1
R2
R3
R4
PSEN1 WT
PSEN1 E280A
VAChT
ChAT
Q1
0,023%
Q2
76,1%
Q4
0,046%
Q3
23,8%
Q1
0,015%
Q2
77,0%
Q4
0,505%
Q3
22,5%
Q1
0,023%
Q2
76,5%
Q4
0,00%
Q3
23,5%
Q1
0,00%
Q2
79,2%
Q4
0,099%
Q3
20,7%
Q1
0,00%
Q2
75,8%
Q4
0,00%
Q3
24,2%
Q1
0
Q2
78,7
Q4
0
Q3
21,3
Unstained Cells
WT ChLNs
E280A ChLNs

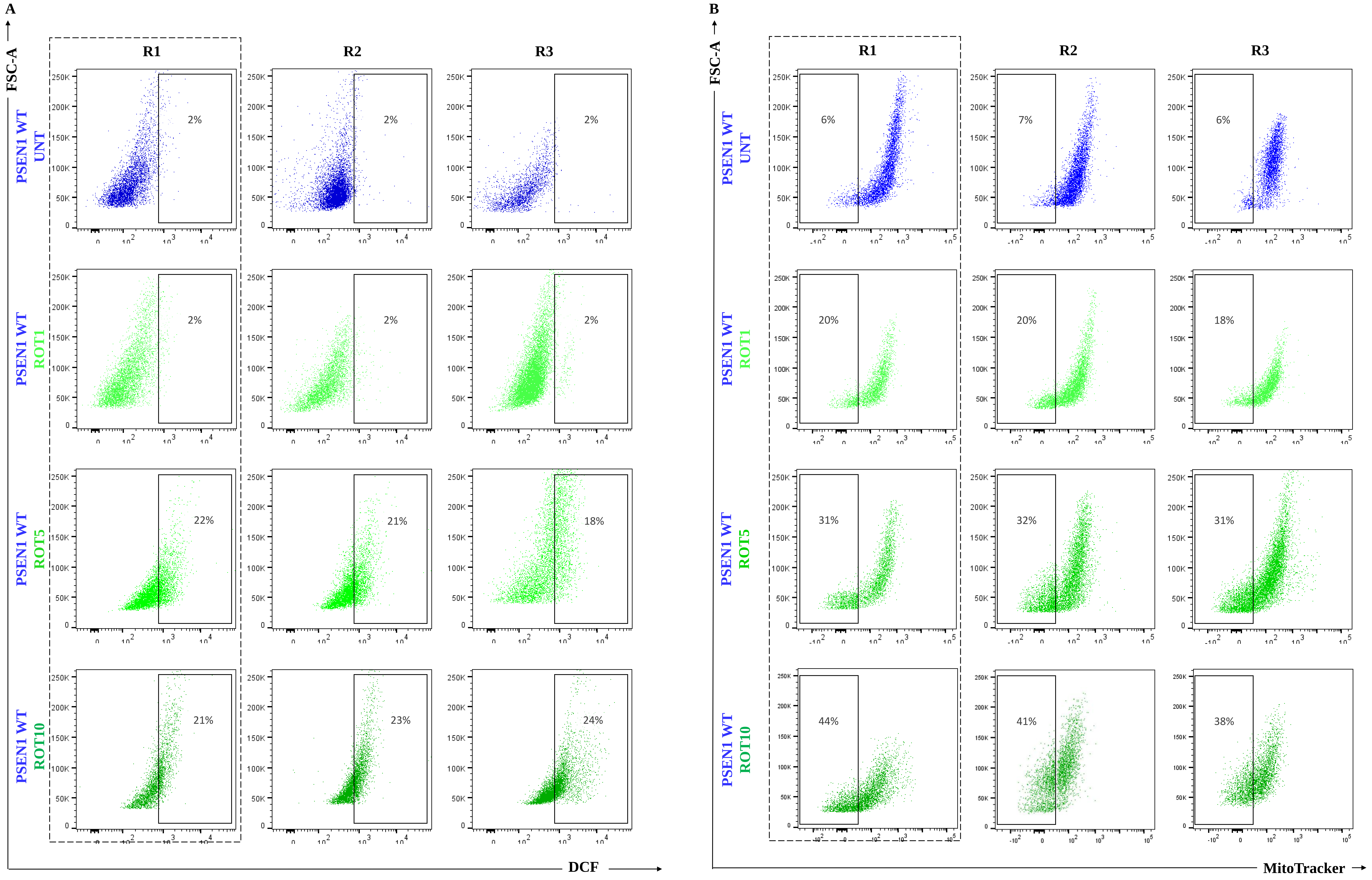

Supplementary Figure 2

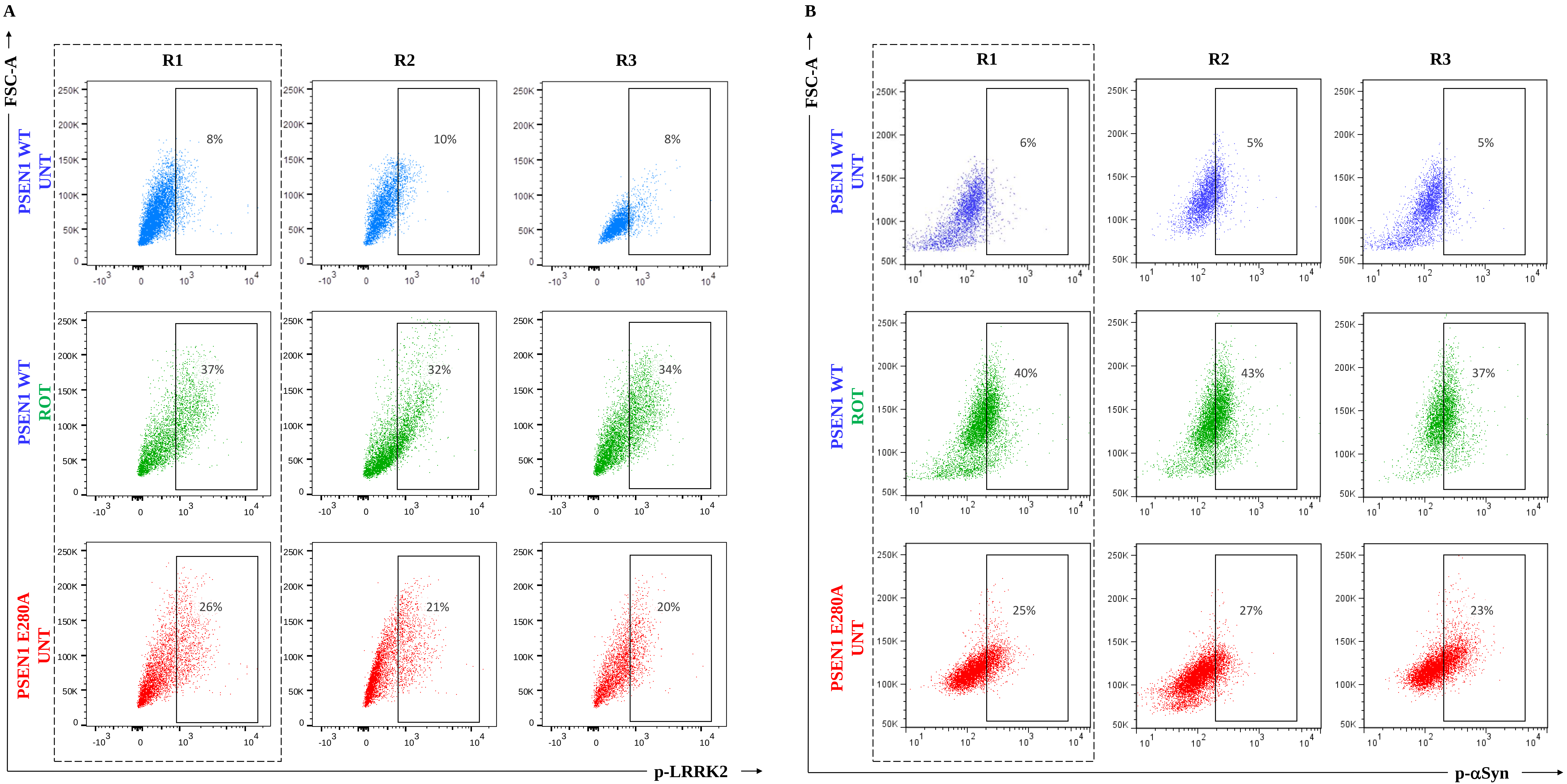

Supplementary Figure 3

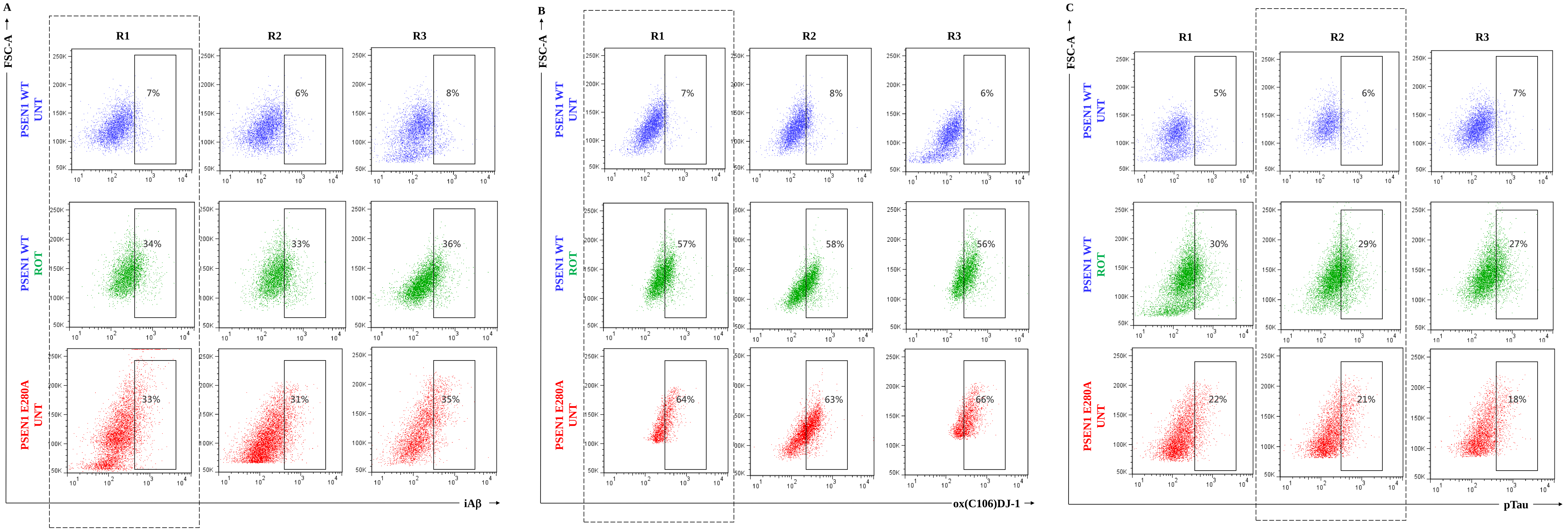

Supplementary figure 4

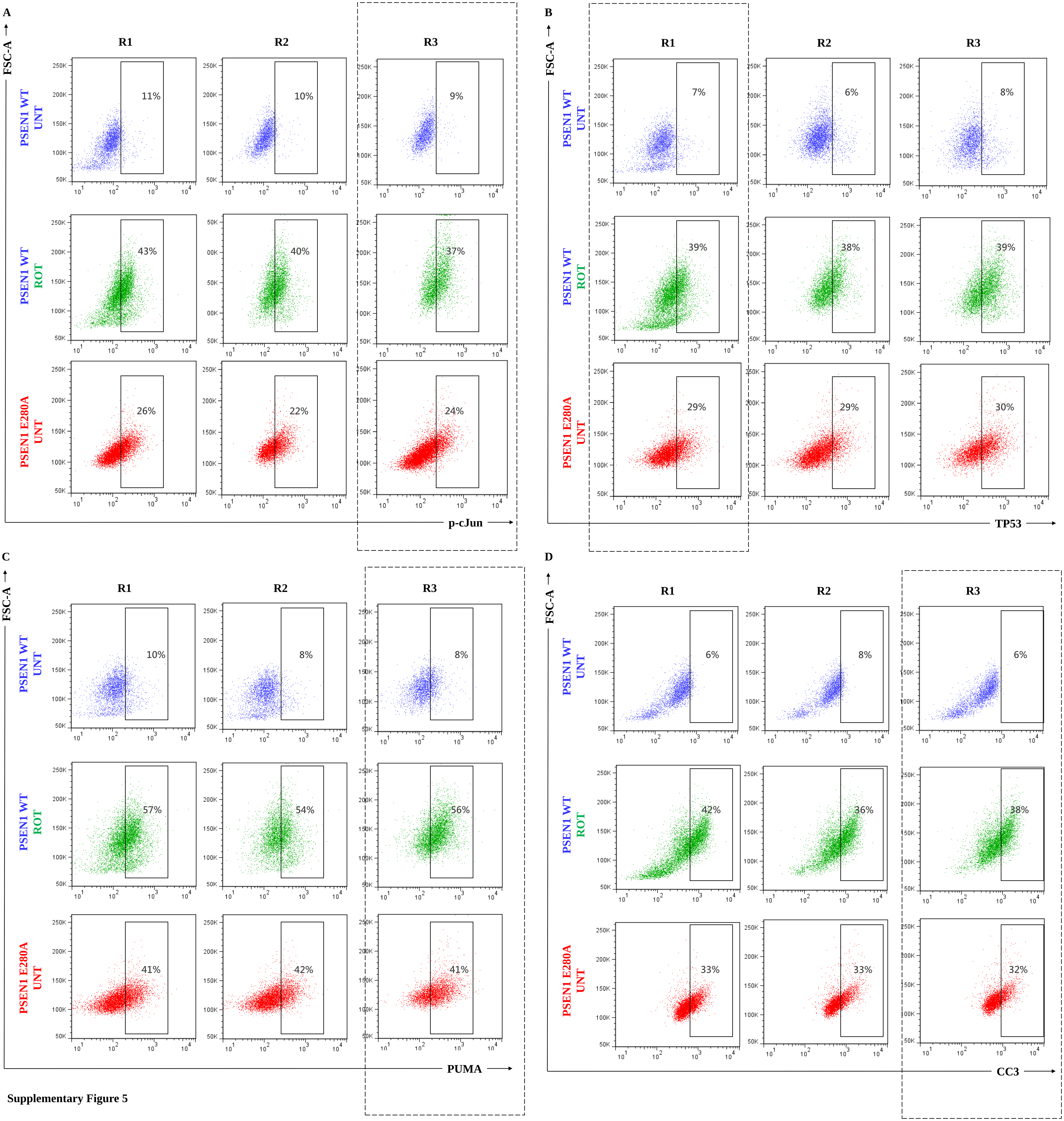

Supplementary Figure 5

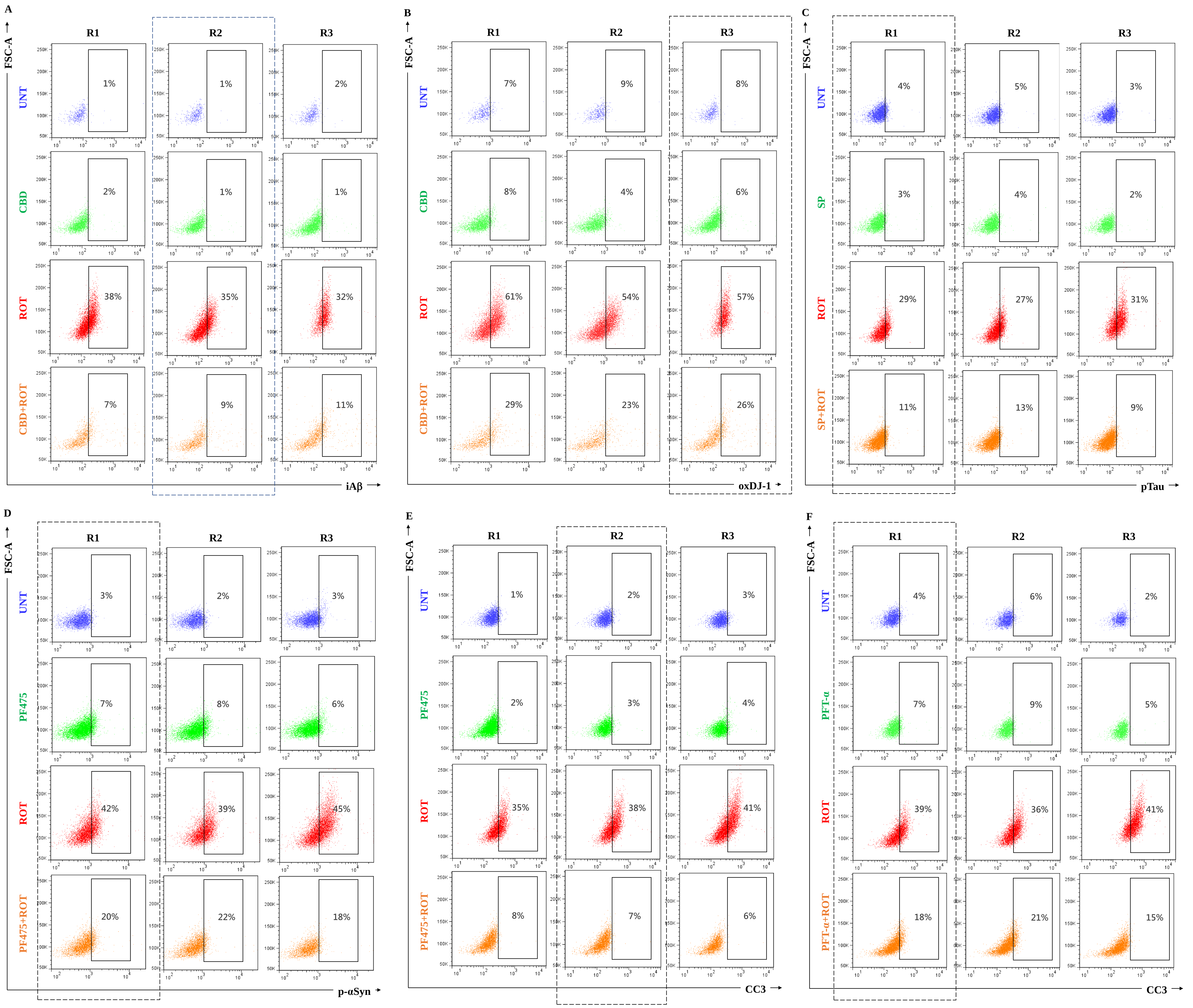

Supplementary figure 6
